# FoQDE2-dependent milRNA promotes *Fusarium oxysporum* f. sp. *cubense* virulence by silencing a glycosyl hydrolase coding gene expression

**DOI:** 10.1371/journal.ppat.1010157

**Published:** 2022-05-05

**Authors:** Minhui Li, Lifei Xie, Meng Wang, Yilian Lin, Jiaqi Zhong, Yong Zhang, Jing Zeng, Guanghui Kong, Pinggen Xi, Huaping Li, Li-Jun Ma, Zide Jiang

**Affiliations:** 1 Department of Plant Pathology / Guangdong Province Key Laboratory of Microbial Signals and Disease Control, South China Agricultural University, Guangzhou, PR China; 2 Department of Biochemistry and Molecular Biology, University of Massachusetts, Amherst, Massachusetts, United States of America; 3 Bioinformatics section, National Institute of Neurological Disorders and Stroke, NIH, Bethesda, Maryland, United States of America; Institute of Microbiology, Chinese Academy of Sciences, CHINA

## Abstract

MicroRNAs (miRNAs) are small non-coding RNAs that regulate protein-coding gene expression primarily found in plants and animals. Fungi produce microRNA-like RNAs (milRNAs) that are structurally similar to miRNAs and functionally important in various biological processes. The fungus *Fusarium oxysporum* f. sp. *cubense* (*Foc*) is the causal agent of Banana *Fusarium* vascular wilt that threatens global banana production. It remains uncharacterized about the biosynthesis and functions of milRNAs in *Foc*. In this study, we investigated the biological function of milRNAs contributing to *Foc* pathogenesis. Within 24 hours post infecting the host, the Argonaute coding gene *FoQDE2*, and two Dicer coding genes *FoDCL1* and *FoDCL2*, all of which are involved in milRNA biosynthesis, were significantly induced. *FoQDE2* deletion mutant exhibited decreased virulence, suggesting the involvement of milRNA biosynthesis in the *Foc* pathogenesis. By small RNA sequencing, we identified 364 small RNA-producing loci in the *Foc* genome, 25 of which were significantly down-regulated in the *FoQDE2* deletion mutant, from which *milR-87* was verified as a FoQDE2-depedent milRNA based on qRT-PCR and Northern blot analysis. Compared to the wild-type, the deletion mutant of *milR-87* was significantly reduced in virulence, while overexpression of *milR-87* enhanced disease severity, confirming that *milR-87* is crucial for *Foc* virulence in the infection process. We furthermore identified *FOIG_15013* (a glycosyl hydrolase-coding gene) as the direct target of *milR-87* based on the expression of FOIG_15013-GFP fusion protein. The *FOIG_15013* deletion mutant displayed similar phenotypes as the overexpression of *milR-87*, with a dramatic increase in the growth, conidiation and virulence. Transient expression of FOIG_15013 in *Nicotiana benthamiana* leaves activates the host defense responses. Collectively, this study documents the involvement of milRNAs in the manifestation of the devastating fungal disease in banana, and demonstrates the importance of milRNAs in the pathogenesis and other biological processes. Further analyses of the biosynthesis and expression regulation of fungal milRNAs may offer a novel strategy to combat devastating fungal diseases.

## Author summary

The fungus *Fusarium oxysporum* f. sp. *cubense* (*Foc*) is the causal agent of Banana *Fusarium* vascular wilt that threatens global banana production. However, knowledge about pathogenesis of *Foc* is limited. In particular, pathogenic regulatory mechanism of the microRNA like small RNAs (milRNAs) found in *Foc* is unknown. Here, we found that *FoQDE2*, an Argonaute coding gene, and two Dicer coding genes *FoDCL1* and *FoDCL2*, which are involved in milRNA biosynthesis, are significantly induced during the early infection stage of *Foc*. The results suggested that the milRNAs biosynthesis mediated by these genes may play an active role in the infection process of *Foc*. Based on this assumption, we subsequently found a FoQDE2-dependent milRNA (*milR-87*) and identified its target gene. Functional analysis showed that *FoQDE2*, *milR-87* and its target gene were involved in the pathogenicity of *Foc* in different degree. The studies help us gain insight into the pathogenesis with *FoQDE2*, *milR-87*, and its target gene as central axis in *Foc*. The identified pathogenicity-involved milRNA provides an active target for developing novel and efficient biocontrol agents against Banana *Fusarium* wilt.

## Introduction

Banana *Fusarium* wilt, also known as Panama disease, is caused by the fungal pathogen *Fusarium oxysporum* f. sp. *cubense* (*Foc*). And it poses a serious threat to the banana industry worldwide [1,2]. Four physiological races of *Foc* have been identified, of which tropical race 4 (TR4) has the most devastating effect on banana production [2,3]. TR4 originated in Southeast Asia and is the main cause of banana *Fusarium* wilt in China [3–6]. TR4 has spread rapidly around the world, and has been reported in Mozambique, Australia, Pakistan, and even in countries along the Mediterranean coast, in countries such as Lebanon, Oman, and Jordan [1,2,7]. However, fungicides, flood fallowing, and organic amendments have rarely provided long-term control in any banana planting area. The only effective method for controlling the dissemination and subsequent infections by *Foc* in banana is by the quarantine or exclusion of infected properties or by planting non-host crops or cultivars [8]. Lack of effective methods to control banana fusarium wilt seriously imperils global banana production. Improved strategies to control this devastating disease are urgently needed.

MicroRNAs (miRNAs), a type of small non-coding single-stranded RNAs, play crucial roles in diverse biological processes [9,10]. Through base pairing with target messenger RNAs (mRNAs), miRNAs degrade the target mRNA or inhibit its translation and thereby regulate gene expression at the post transcription level [11]. Small RNAs (sRNAs) have been reported in various fungi, including *Neurospora crassa*, *Magnaporthe oryzae*, *Botrytis cinerea*, and *Sclerotinia sclerotiorum* [12–15]. Because some of these sRNAs are structurally similar to miRNAs from plants and animals, they are called microRNA-like RNAs (milRNAs) [12,14]. In *B*. *cinerea*, milRNAs have been identified as virulent effectors that suppress host immunity and facilitate fungal infection [14,16]. In *Verticillium dahliae*, a novel milRNA, VdmilR1, was reported to play crucial roles in pathogenicity [17]. Recently, a milRNA (*Vm*-milR37) expressed exclusively in the mycelium, was verified to contribute to pathogenicity in *Valsa mali* [18]. On the other hand, plants also export miRNAs or siRNAs to inhibit gene expression in fungal pathogens and confer efficient crop protection from pathogen infection [19–21]. Thus, the trans-kingdom sRNAs play key roles in host-pathogen interactions [22].

Four types of milRNAs generated from four different biosynthesis pathways, namely milR-1 to -4, have been reported in the model fungus *N*. *crassa* [12]. Different combinations of factors, including Dicers, QDE2 (Quelling Deficient 2), the exonuclease QIP (QDE2 interacting protein), and an RNAse III domain-containing protein, MRPL3, are involved in the production of milRNAs [12,23,24]. The reported milRNA biosynthesis pathways in *N*. *crassa* appear more complex and diverse than that in plants and animals [25].

Argonaute (AGO) proteins are evolutionarily conserved in all domains of life and play a key role in the RNA interference (RNAi) pathway [26]. As in plants and animals, AGO proteins are the core components of the RNA-induced silencing complex (RISC) and contribute to gene silencing by using the loaded sRNA as a specificity determinant in fungi [15]. QDE2, one of the two identified AGO proteins in *N*. *crassa*, functions as a slicer and is required for the biogenesis of some sRNAs such as milRNAs and PIWI-interacting RNAs [12,24]. Suppressor of meiotic silencing 2 (Sms-2), another reported AGO protein in the *N*. *crassa* genome, is thought to function by binding to sRNAs originating from the unpaired DNA region and is required for meiotic silencing of unpaired DNA [25,27]. In the model fungus *N*. *crassa*, milR-1 type of milRNAs are the most abundant milRNAs and the maturation of the milRNAs requires the AGO protein QDE-2 [12,24]. However, whether this type of milRNAs exist and what function they have in *Foc* remain unclear.

In this study, we identified an AGO and two Dicer proteins in *Foc*, and examined their functions in milRNAs biosynthesis and in fungal pathogenesis by sRNA sequencing and reverse genetics. We identified a FoQDE2-dependent milRNA (*milR-87*), which contributes to invasive growth during the initial stage of *Foc* infection and thus affects *Foc* pathogenicity, likely by targeting the gene *FOIG_15013*. *FOIG_15013* encodes a glycosyl hydrolase, which appears as a negative regulator of *Foc* conidiation and pathogenicity. Overall, our findings uncover the novel function of milRNA in *Foc* pathogenicity.

## Results

### Identification of AGO protein FoQDE2 in *Foc*

**Fig 1:**
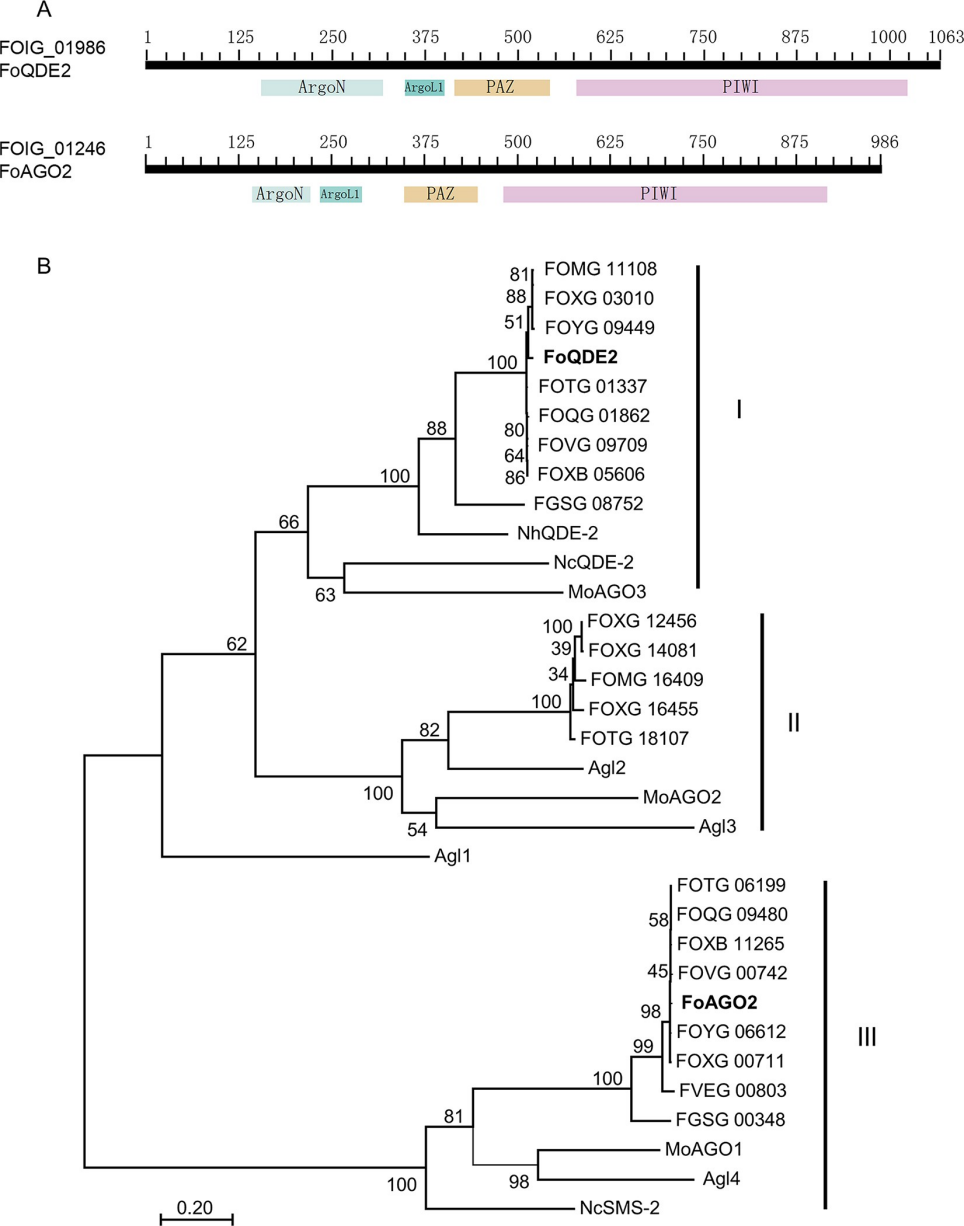
Phylogenetic analysis of fungal Argonaute proteins. (**A**) Conserved domains of Argonaute proteins were predicted through BLASTp on NCBI. (**B**) Fungal Argonaute protein sequences were first aligned using Clustal×1.8 and then the aligned sequences were analyzed by the Maximum Likelihood method implemented in MEGA 7. Bootstrap values are expressed as a percentage of 1000 replicates. In addition to Argonaute proteins from the *Fusarium oxysporum* f. sp. *cubense* (*Foc*) genome showed in bold, proteins used for this analysis include Agl1–Agl4 from *Cryphonectria parasitica* [28], a chestnut blight fungus; MoAGO1–MoAGO3 from *Magnaporthe oryzae*, a rice blast fungus [15]; NcQDE2 and NcSMS-2 from the model fungus *Neurospora crassa* [25]; and proteins from the *F*. *graminearum* (FGSG), *F*. *verticillioides* (FVEG), and *F*. *oxysporum* (FOVG: GCA_000260075.2; FOQG: GCA_000260235.2; FOTG: GCA_000260175.2; FOMG: GCA_000260495.2; FOXG: GCA_000149955.2; and FOYG: GCA_000271745.2) genomes.

By performing an orthologous protein BLAST (Basic Local Alignment Search Tool, https://blast.ncbi.nlm.nih.gov/Blast.cgi) search, we identified two AGO proteins in *Foc*, encoded by *FoQDE*2 (FOIG_01986) and *FoAGO2* (FOIG_01246) respectively. Conserved domain prediction showed that the proteins have four domains: a variable N-terminal domain (ArgoN), a linker 1 domain (ArgoL1), a PAZ domain, and a PIWI domain (Fig 1A).

The amino acid sequences of AGO orthologs in other fungal species were retrieved from GenBank for phylogenetic analysis. Two to five AGO-like proteins were identified in different strains of *F*. *oxysporum*, but only two in *Foc*. Phylogenetic analysis indicated that AGO proteins from filamentous fungi could be divided into three subgroups (Fig 1B). In the first group, *FoQDE2* and its orthologs from *F*. *oxysporum* and *F*. *graminearum* were clustered with QDE2 from *N*. *crassa*, AGO-like protein MoAGO3 from *M*. *oryzae* [15], and Agl1 from the chestnut blight fungus *Cryphonectria parasitica* [28]. The second group includes AGO-like proteins from *F*. *oxysporum* and Agl2 and Agl3 from *C*. *parasitica*. The third group is composed of FoAGO2 and its orthologs from *F*. *oxysporum* and *F*. *graminearum*, Agl4 from *C*. *parasitica*, MoAGO1 from *M*. *oryzae*, and SMS2 from *N*. *crassa* [25]. The phylogenetic analysis showed that the AGO proteins in *Foc* are orthologous to those of the other filamentous fungi.

### Characterization of sRNA biosynthesis-related genes in *Foc*

**Fig 2:**
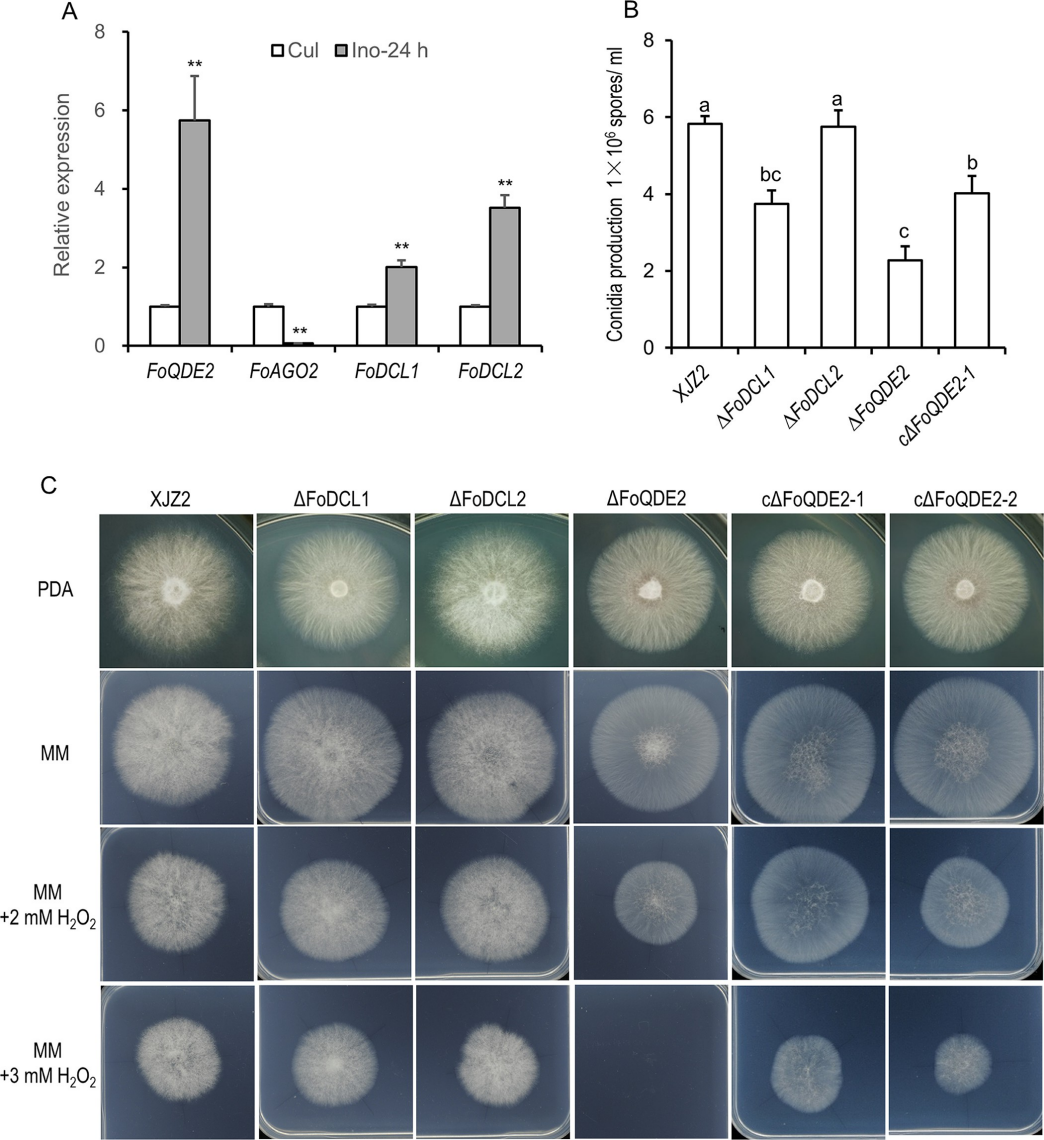
Characterization of milRNA biosynthetic pathway genes in *F*. *oxysporum* f. sp. *cubense*. (**A**) Expression patterns of milRNA biosynthetic pathway genes at pure culture conditions (Cul) and 24 hours post inoculation (Ino-24h). Relative folds were calculated by 2^-ΔΔCt^ method [56], using transcription elongation factor 1 α gene (*EF1α*) as internal control. Error bars indicate S.D. (n = 3). A Student’s *t*-test was used for significant analysis. **, p<0.01. (**B**) Conidial production of the tested strains. The tested strains were grown on PDA plates at 28°C for 7 days. Then same amounts of colony discs were washed by water supplemented with 0.05% tween 20 to prepare conidia suspension. And conidia were quantified under microscope using a haemocytometer. A Duncan’s multiple range test was used for significant analysis. Error bars indicate S. D. (n = 10). Different letters indicate significant difference at the level of α = 0.01. (**C**) Colony morphology of the tested strains grown on PDA, minimal medium (MM), MM supplemented with 2 or 3 mM H_2_O_2_. The wild-type strain XJZ2, milRNA biosynthetic pathway gene knockout mutants (Δ*FoQDE2*, Δ*FoDCL1*, and Δ*FoDCL2*), and two *FoQDE2* complementation transformants (cΔ*FoQDE2*-1 and cΔ*FoQDE2*-2) were inoculated on PDA and MM with 0, 2, or 3 mM H_2_O_2_ and were cultured at 28°C. Colony morphology and mycelial growth were recorded after 3 days of culture.

In addition to the AGO coding genes, two Dicer coding genes (*FoDCL1* and *FoDCL2*) exist in *Foc*, which function in the biosynthesis of fungal sRNAs [12]. Compared with *Foc* in pure culture conditions, *FoQDE2* and Dicer coding genes were significantly up-regulated at 24 hours post inoculation (hpi) on host banana plants. In contrast, transcription of *FoAGO2* was nearly undetectable (Fig 2A). The transcriptional induction of these sRNA biosynthesis genes during host infection stage suggests that the sRNAs synthesis mediated by these genes may play an active role during *Foc* pathogenesis.

To further investigate the function of the three up-regulated genes (*FoQDE2*, *FoDCL1*, and *FoDCL2*), we generated these genes deletion mutants in the wild-type (WT) strain XJZ2 via homologous recombination (S1A Fig). Four independent *FoQDE2-*deletion mutants were verified by PCR and Southern blot analysis (S1B and S1C Fig). Four *FoDCL1-*deletion mutants and *FoDCL2-*deletion mutants were respectively obtained and verified by Southern blot analysis (S1C Fig). Four complemented strains were also generated by expressing the *FoQDE2* locus (S1A Fig) in one of the Δ*FoQDE2* mutant, as confirmed by PCR (S1D Fig) with primer pairs FoQDE2-F1/FoQDE2-R, HYG-F/HYG-R2, and Zeo-F/Zeo-R (position of the primer pairs were presented in S1A Fig; sequences listed in S1 Table).

Relative transcript levels of *FoQDE2*, *FoDCL1*, and *FoDCL2* were examined by quantitative real-time PCR (qRT-PCR) analysis in the WT strain and the mutants. No *FoQDE2*, *FoDCL1*, or *FoDCL2* transcript was detected in the corresponding deletion mutants (S2A Fig), confirming that the genes had been successfully deleted in the respective mutant. Transcript level of *FoQDE2* was detected in the complemented strains, to a comparable level of that in the WT (S2B Fig), indicating that *FoQDE2* was restored in the complemented strains (cΔ*FoQDE2*-1/-2).

### Characterization of FoQDE2 during growth, conidial production, stress tolerance and virulence to banana seedlings in *Foc*

We next assessed the biological function of FoQDE2 by examining growth and conidiation phenotypes in the Δ*FoQDE2* mutants. The results showed that, the colonial morphology of the Δ*FoQDE2* was strikingly different from that of the WT strain XJZ2. The aerial hyphae of Δ*FoQDE2* were much less abundant than those of the WT, and the mycelia grew close to the surface of the PDA media. Such morphological change of the colonies was restored to different degrees in the two complemented *FoQDE*2 strains (cΔ*FoQDE2*-1 and cΔ*FoQDE2*-2; Fig 2C). On the other hand, the colony morphology was not much changed in the Δ*FoDCL1* or Δ*FoDCL2* mutants (Fig 2C).

We assessed mycelial growth rate in the WT and mutant strains. From the fourth day of culture, the growth of Δ*FoQDE2* was significantly slower than that of the WT at the level of α = 0.01. The growth of the complemented strains was not fully recovered. The Δ*FoDCL1* and Δ*FoDCL2* mutants displayed no difference in mycelial growth compared with the WT (S2 Table). Thus, loss of *FoQDE2* led to slow mycelial growth on PDA medium.

Furthermore, microconidia production was significantly reduced in the Δ*FoQDE2* mutant, compared to the WT, when cultured on PDA medium for 7 days. Such conidiation defect could be partially restored in the *FoQDE2* complemented strains (Fig 2B). Compared with the WT, Δ*FoDCL1* also produced less microconidia, whereas Δ*FoDCL2* showed no significant difference in conidial production (Fig 2B).

To elucidate the causes of the decreased conidia production in Δ*FoQDE2*, six reported conidiation-related genes [29–32] were selected for their transcript levels assessment by qRT-PCR. Compared with the WT, the transcript levels of four selected conidiation-related genes, *StuA*, *FoNIIA*, *AbaA*, and *WetA*, were significantly down-regulated in Δ*FoQDE*2. Transcription of *StuA* and *FoNIIA* were restored in the *FoQDE2* complemented strain (cΔ*FoQDE2*-1), to a level comparable to the WT (S2C Fig). The transcript levels of *brlA* and *Htf1* were not affected by the loss of *FoQDE2* (S2C Fig). Overall, our results showed that deletion of *FoQDE2* leads to morphological changes, including changes in colonial morphology and reduction of mycelial growth, as well as reduction in conidial production of *Foc*.

We next tested the sensitivities of the WT and the mutant strains to oxidative stress. The Δ*FoQDE2* mutant was hypersensitive to 3 mM H_2_O_2_ when cultured on minimal medium (MM), which could be restored in the complemented strains (Figs 2C and S2D). However, the Δ*FoDCL1* and Δ*FoDCL2* mutants showed no difference in sensitivity to oxidative stress as compared to the WT when cultured on MM supplemented with 2 or 3 mM H_2_O_2_ (Figs 2C and S2D). Taken together, FoQDE2 is required for tolerance to oxidative stress.

**Fig 3:**
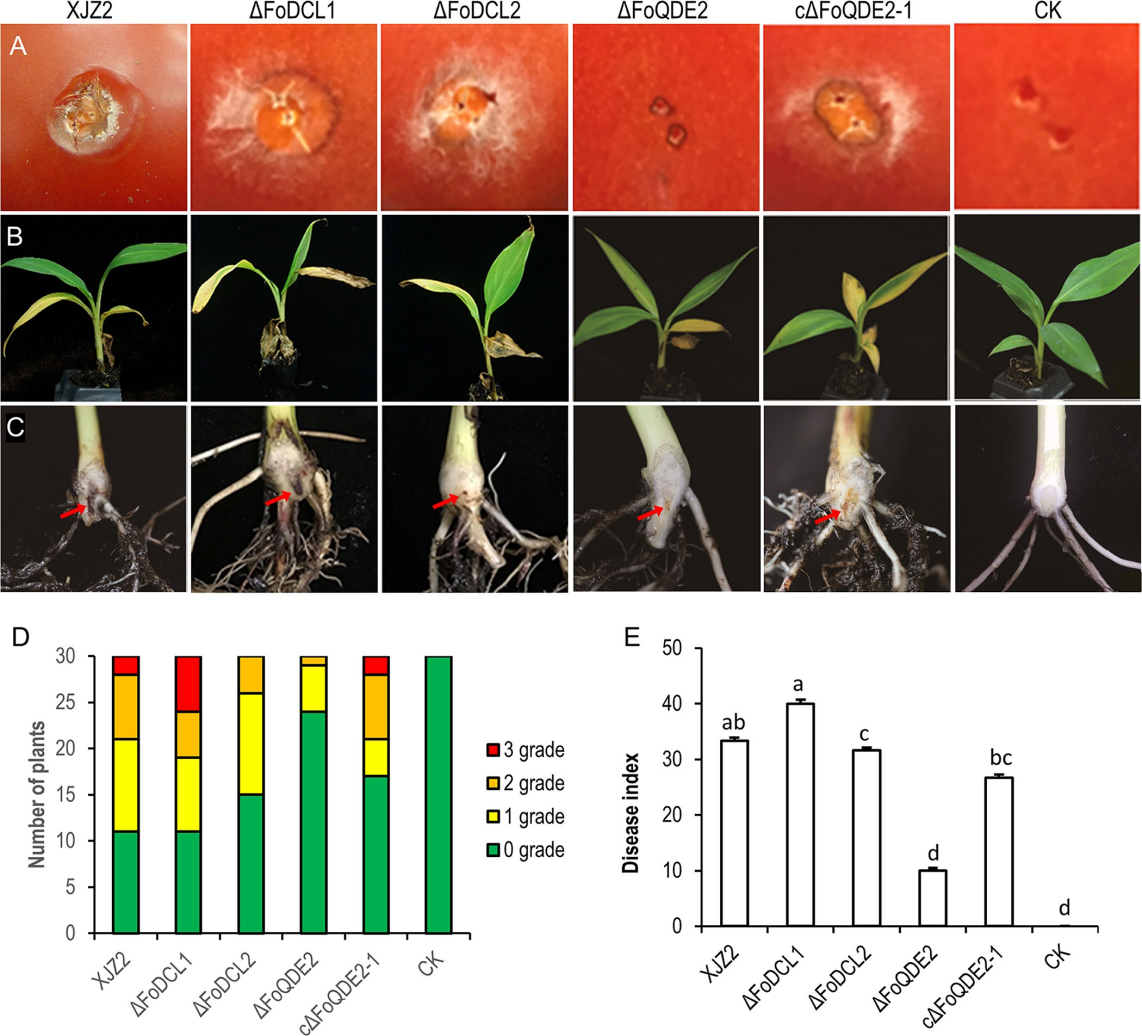
Pathogenicity assay. (**A**) Invasive growth on tomato fruits. The surfaces of tomato fruits were inoculated with the WT strain XJZ2, Δ*FoQDE2* mutant, the cΔ*FoQDE2*-1 complementation transformant, and water control (CK). The incidence of necrosis on tomato fruits was assessed at 5 days post inoculation. (**B**) and (**C**) Symptoms of pathogenicity on the leaves (B) and corm (C) of the banana plant. (**D**) and (**E**) Disease severity analyzed by diseased plantlet number of different disease grade (D) and disease index (E). The disease grade was classified as 0 (no symptoms in the corm of the banana plantlet), 1 (the presence of small vascular discoloration like brown dots), 2 (up to 50% of vascular discoloration) and 3 (greater than 50% of vascular discoloration). Different letters indicate significant difference at the level of α = 0.01.

Mycelial infection capacity was tested on the tomato fruit surface and the incidence of tomato fruit infection was recorded at five days post inoculation (dpi). The Δ*FoQDE2* mutant did not penetrate the epidermis or cause fruit tissue necrosis on the surface of tomato as the WT and complemented strains did (Fig 3A). In contrast, the Δ*FoDCL1* and Δ*FoDCL2* mutants caused tissue necrosis similar to that of WT (Fig 3A). Thus, FoQDE2 is required for successful invasive growth.

Pathogenicity tests on banana (Cavendish) seedlings showed that the Δ*FoQDE2* mutant was unable to produce obvious vascular discoloration in the corm of banana seedling (Fig 3B and 3C) and was significantly reduced in virulence on banana compared to the WT (*p*<0.01, Fig 3D and 3E). The disease index of the complemented strain (cΔ*FoQDE2*-1) was increased but did not reach the level of the WT (Fig 3E), which exhibited brown discoloration in the corm of banana seedlings. On the other hand, compared to the WT, pathogenicity of the Δ*FoDCL1* mutant was not changed, while pathogenicity of the Δ*FoDCL2* mutant was decreased significantly (Fig 3D and 3E). These observations indicate that *FoQDE2* is involved in the virulence of *Foc*.

### Identification of FoQDE2-dependent milRNA in *Foc*

**Fig 4:**
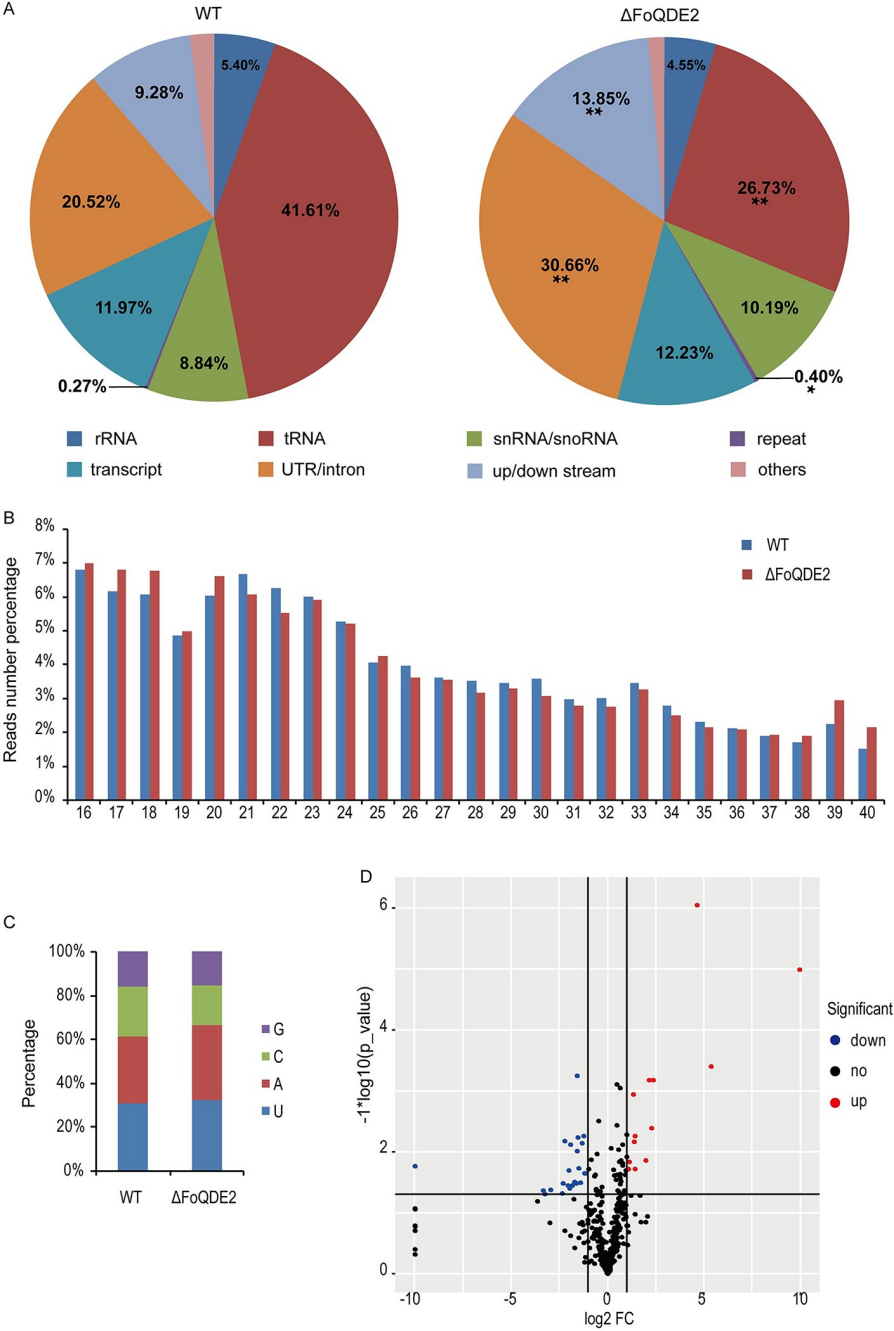
Small RNA sequencing analysis in *F*. *oxysporum* f. sp. *cubense*. (**A**) Characterization of small RNA-producing loci. (**B**) Length distribution of small RNAs. (**C**) Analysis of the 5′ end nucleotide preference of small RNAs. (**D**) Differential analysis of small RNA expression in the WT and Δ*FoQDE2* mutant.

To investigate the function of FoQDE2 in sRNA production, we sequenced sRNAs from the WT strain and Δ*FoQDE2* mutant. The sequencing data were deposited in the NCBI sequence read archive (SRA) under accession number PRJNA562097. Origin analysis of the sRNAs showed that reads mapped to tRNA in Δ*FoQDE2* was significantly reduced by approximately 40% than in the WT, whereas significantly more reads mapped to the UTRs, intronic, and intragenic regions in Δ*FoQDE2* than in the WT (Fig 4A). sRNA length distribution analysis showed that most reads with length of between 16–24 nt were in both WT and Δ*FoQDE2* mutant, while reads of 21 and 22 nt were reduced in the Δ*FoQDE2* mutant compared to the WT (Fig 4B). Reads starting with A or U were more abundant than those starting with C or G (Fig 4C). To identify sRNA-producing loci, reads with counts of 10 or higher that mapped to the UTR and intronic and intergenic regions were analyzed. Overlapping and adjacent reads were grouped and only those with a consensus length of less than 300 nt were considered to be small RNA-producing loci. Using this method, 364 loci were captured in the WT and Δ*FoQDE2* mutant. Among them, 25 loci were significantly decreased and 13 loci were significantly increased in Δ*FoQDE2* compared with the WT. (Fig 4D). A total of 12 sRNAs coming from these loci were selected for expression verification through reverse transcription-PCR. And the results of electrophoresis showed that the expression of selected sRNAs was consistent with the results of sequencing when using small nuclear RNA U4 of *Foc* as the internal control (S3A and S3B Fig).

**Fig 5:**
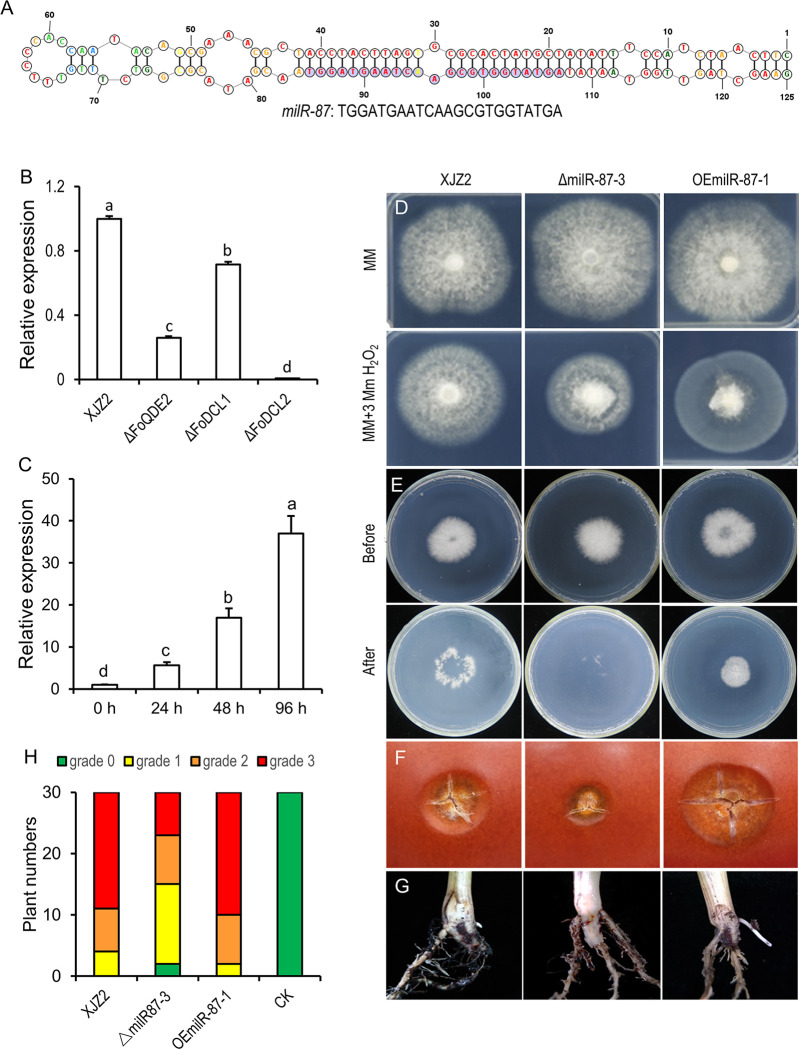
Characterization of FoQDE2-dependent milRNA (*milR-87*) in *F*. *oxysporum* f. sp. *cubense* (*Foc*). (**A**) Predicted secondary structure of *milR-87* precursor in *Foc*. (**B**) *MilR-87* expression in different milRNA biosynthetic pathway gene deletion mutants (Δ*FoQDE2*, Δ*FoDCL1*, and Δ*FoDCL2*) by qRT-PCR. Error bars indicate S.D. (n = 3). Different letters indicate significant difference at the level of α = 0.01. (**C**) Expression patterns of *milR-87* at 0, 24, 48, and 96 hours post inoculation. Total RNAs were extracted from the host banana roots inoculated with *Foc* at the different time point (0–96 h). Relative expression levels of *milR-87* were calculated by 2^-ΔΔCt^ method using snRNA (U4) as internal control. Error bars indicate S. D. (n = 3). Different letters indicate significant difference at the level of α = 0.01. (**D**) Mycelial sensitivity to H_2_O_2_. Mycelial growth of the WT strain XJZ2, the Δ*milR-87* mutant (Δ*milR-87-3*) and *milR-87* over-expression strain OEmilR-87-1 were measured at MM with 0 and 3 mM H_2_O_2_. (**E**) Cellophane penetration assay comparing the invasive growth of the XJZ2, Δ*milR-87* mutant and OEmilR-87 strain. Conidia suspensions (100 μl per strain) with same concentration of 1×10^5^ spores/ml were put on cellophane-covered PDA plates and incubated at 28°C for 4 days (Before, before cellophane removed), then the cellophane sheets were removed, and samples were incubated for an additional 3 days (After, after cellophane removed) and the colony size indicated mycelial growth were photographed. (**F**) Invasive growth on tomato fruits. (**G**) Symptoms of pathogenicity on the corm of the host banana seedling. (**H**) Disease severity analyzed by diseased plantlet number of different disease grade.

To identify FoQDE2-dependent milRNA, sRNA read counts obtained from the WT and Δ*FoQDE2* were normalized and compared. To eliminate interference from sRNAs produced by mRNA degradation, the reads mapped to ORFs were discarded. A total of 25 sRNA-producing loci were significantly decreased in Δ*FoQDE2* compared with the WT. Sequences of the loci were extracted from the *Foc* II5 genome and uploaded to the RNAfold web server (http://rna.tbi.univie.ac.at//cgi-bin/RNAWebSuite/RNAfold.cgi) for RNA advanced structure prediction. The loci with a stem-loop RNA structures were validated as precursors of milRNAs. Among them, four milRNAs were predicted to form stem-loop structures. Three of them (milRNA loci were marked as INFOIG_12093, DSFOIG_07638 and DSFOIG_15767) were confirmed with down-regulated expression in the Δ*FoQDE2* mutant by reverse transcription-PCR (S3A Fig). We failed to detect expression of the fourth one (UTRFOIG_14681) for its rich in Adenine and Uracil nucleotides. In this study, we focused on one milRNA (milR-87 located at the downstream of *FOIG_15767*, DSFOIG_15767) that formed a stem-loop precursor (Fig 5A). The mature *milR-87* was predicted to be of 23 nt (Fig 5A). T-clone and sequencing of *milR-87* confirmed the results. We further verified expression level of *milR-87* using qRT-PCR (Fig 5B) and sRNA Northern blot (S4E Fig). Compared to the WT, the production of *milR-87* was significantly reduced in the Δ*FoQDE2* and Δ*FoDCL2* mutants (Figs 5B and S4E). Based on our sRNA analysis, *milR-87* is dependent on FoQDE2 and FoDCL2 in *Foc*.

### MilRNA (*milR-87*) regulates tolerance to hydrogen peroxide and is involved in the virulence of *Foc*

To determine the function of the *milR-87*, we generated deletion or overexpression mutants of *milR-87* precursor in the WT *Foc* strain (S4A Fig). PCR results showed that the *milR-87* precursor was successfully deleted or overexpressed in *Foc* (S4B and S4C Fig). Northern blot and qRT-PCR results confirmed that the *milR-87* was deleted or overexpressed in the corresponding mutants (S4D and S4E Fig).

The Δ*milR-87* mutant was hypersensitive to the oxidative stress generated by 3 mM H_2_O_2_, a phenotype similar to that of the Δ*FoQDE2* mutant (Fig 5D). In contrast, the growth of *milR-87* overexpress transformant (OEmilR-87-1) was not affected by oxidative stress (Fig 5D). The result indicates *milR-87* positively regulates tolerance to oxidative stress in *Foc*.

Pathogenicity tests showed that the Δ*milR-87* mutant was compromised in penetrating the cellophane membrane and caused slighter necrosis on the surface of tomato fruits than the WT. While overexpressed transformant showed the opposite (Fig 5E and 5F). Infection assay using banana seedlings showed that Δ*milR-87* significantly reduced in virulence compared to the WT, while the virulence of overexpressed transformant was enhanced, as it caused obvious internal disease symptoms of brown discoloration (Fig 5G and 5H). Furthermore, qRT-PCR analysis showed that *milR-87* was significantly up-regulated during the early stages of infection (24, 48, and 96 hpi) compared with levels at 0 hpi conditions (Fig 5C). These results suggest that *milR-87* contributes to the infective growth and virulence of *Foc*.

**Fig 6:**
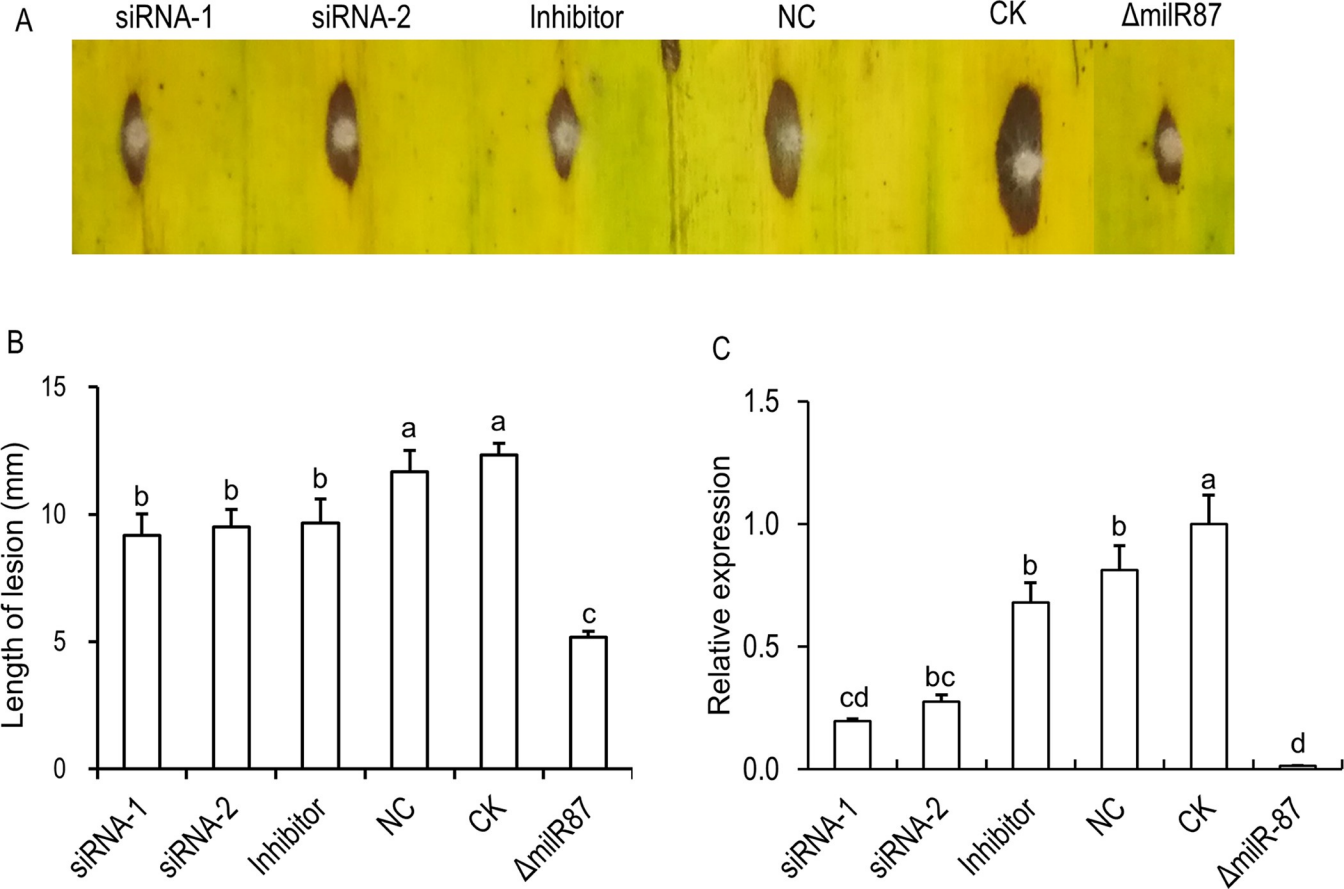
Exogenous siRNAs and inhibitor against *milR-87* attenuated the virulence of *F*. *oxysporum* f. sp. *cubense* to banana leaves. Double-strand siRNAs (siRNA-1 and siRNA-2) and single-strand inhibitor against the precursor sequence of *milR-87*, as well as a random small RNA (24 nt) serving as a negative control (NC) were synthesized and transfected into *Foc* with aid of lipofectamine 2000 (Invitrogen). After 2 days transfection, conidia suspensions of the transfected strains and positive control of the Δ*milR-87* mutant, as well as non-transfected strain of *Foc* (CK) were inoculated on the banana leaves. After 3 days, the lesions on banana leaves were photographed. (**A**) Symptoms of pathogenicity on the host banana leaves. (**B**) Lesions sizes were measured for accessing virulence of the different strains. Error bars indicate S. D. (n = 3). Different letters indicate significant difference at the level of α = 0.01. (**C**) Relative expression of *milR-87* in the transfected strains, the non-transfected strain of *Foc* (CK), and the Δ*milR-87* mutant. The total RNAs of the tested strains cultured at 28°C for 7 days were extracted for milRNA detection by qRT-PCR. Relative expression levels of *milR-87* in the transfected strains were normalized to that of non-transfected strain of *Foc* (CK), which was set as 1. SnRNA U4 was used as internal control. Error bars indicate S. D. (n = 3). Different letters indicate significant difference at the level of α = 0.01.

To confirm the function of milRNA (*milR-87*) in *Foc*, synthetic double-strand siRNAs (siRNA-1 and siRNA-2) and single-strand antisense small RNA (inhibitor) were designed (S1 Table) to silence the expression of *milR-87*. We observed that when the siRNAs or inhibitor was transfected into *Foc* protoplast inoculated on the banana leaf, the size of the lesion decreased significantly compared to those caused by the *Foc* protoplasts with water as control (CK) (Fig 6A and 6B). An unrelated single-strand sRNA served as the negative control (NC) did not interfere with lesion formation when added into the *Foc* protoplasts inoculated on the banana leaf (Fig 6A and 6B). We noticed that the Δ*milR-87* mutant produced the smallest lesion on the banana leaf (Fig 6A and 6B). The qRT-PCR results confirmed that expression of *milR-87* could be silenced by the siRNA-1, siRNA-2 and inhibitor to different extents (Fig 6C). Overall, these results confirmed that *milR-87* is required for full virulence of *Foc*.

### MilRNA (*milR-87*) regulates the virulence by targeting a glycosyl hydrolase gene (*FOIG_15013*) during the pathogenesis of *Foc*

**Fig 7:**
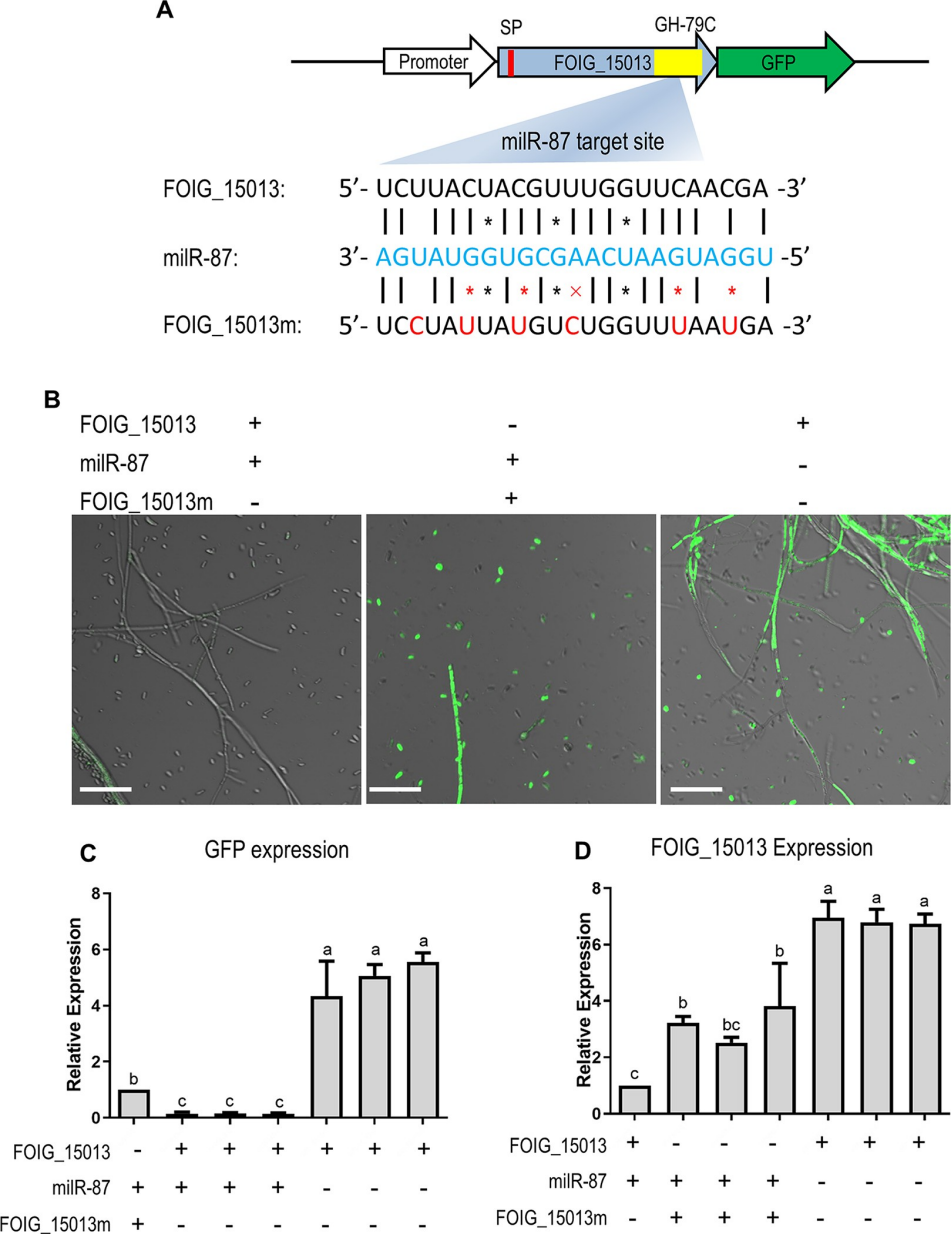
Identification of *milR-87* target gene in *F*. *oxysporum* f. sp. *cubense*. (**A**) Schematic diagram for construct of FOIG_15013-GFP, with the wild-type or mutated (FOIG_15013m) *milR-87* targeted site. The red box represents signal peptide (SP) and the yellow box represents a conserved domain of glycosyl hydrolase 79C (GH-79C) predicted in FOIG_15013. The *milR-87* and its potential target sequence of FOIG_15013 was labeled in blue and black respectively. Point-mutations were in red font. (**B**) Confocal fluorescence microscopic images of the strains expressing FOIG_15013-GFP or FOIG_15013m-GFP, with or without *milR-87*. Bar = 50 μm. (**C**) Transcript level of marker gene GFP in the tested strains. GFP expression values in different strains were normalized to that of point-mutation strain, which GFP value was set as 1. (**D**) Transcript level of *FOIG_15013* in different strains. *FOIG_15013* expression values in different strains were normalized to that of the GFP-labeled WT strain. The transcription elongation factor 1 α gene (*EF1α*) was used as internal control. Error bars indicate S.D. (n = 3). Different letters indicate significant difference at the level of α = 0.01.

To investigate regulatory mechanism of *milR-87* in *Foc* pathogenesis, potential target genes were computationally predicted, and verified by qRT-PCR using total RNAs from the mutants (Δ*milR-87* and Δ*FoQDE2*) and WT. Totally nine genes were significantly up-regulated in the two mutants compared to the WT (S5 Fig). One of these genes, *FOIG_15013*, encodes a glycosyl hydrolase, consisting of a signal peptide and a conserved domain of GH-79C (Fig 7A), was selected for further investigation. The predicted targeted site of *milR-87* was located in the ORF region of *FOIG_15013*, we then generated a fusion protein by directly ligating the GFP coding sequence to the C’-terminus of FOIG_15013 (Fig 7A). A point-mutated FOIG_15013 that could not be paired with *milR-87*, FOIG_15013m, was also fused with GFP (Fig 7A). The constructs were transformed into the Δ*milR-87* mutant or the WT strain respectively, to verify the possible of *milR-87* regulating the FOIG_15013 expression by evaluating GFP signal brightness. In the Δ*milR-87* mutant, FOIG_15013-GFP expression was not inhibited, as indicated by the strong GFP signal (Fig 7B and 7C). In contrast, the GFP signal was hardly detected in the WT strain, likely due to *milR-87* mediated suppression of FOIG_15013-GFP expression (Fig 7B and 7C). The FOIG_15013m-GFP expression was not affected in the WT background, as *milR-87* was unable to pair with the mutation site. Correspondingly, transcript level of *FOIG_15013* and its mutated version in different strains was consistent with that of GFP signal detection (Fig 7D), further confirming that *FOIG_15013* is targeted by *milR-87* in *Foc*.

**Fig 8:**
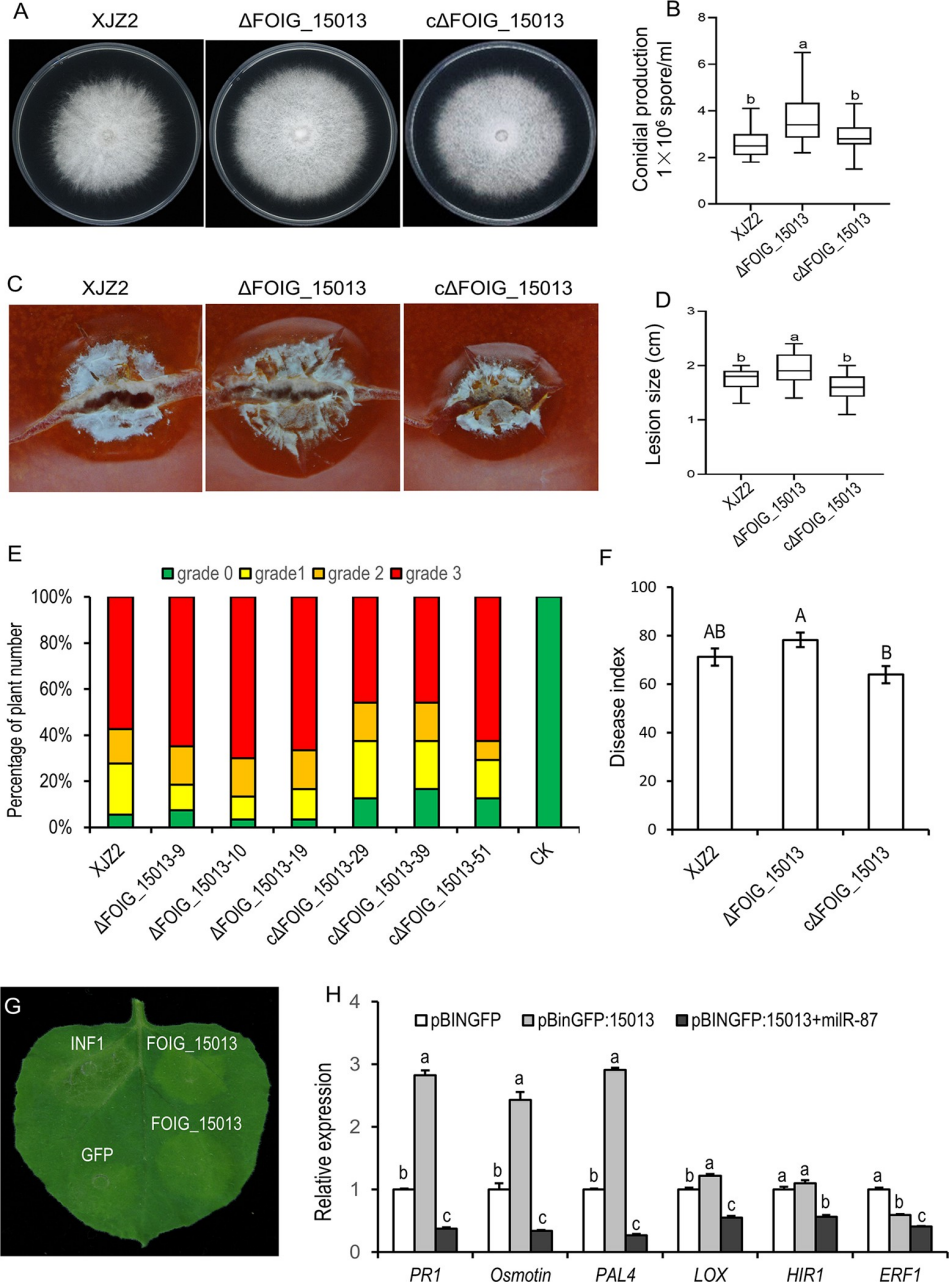
Investigation of FOIG_15013 function in *Foc* growth, conidiation, and pathogenicity. (**A**) Colony morphology of the WT strain XJZ2, *FOIG_15013* deletion mutant (Δ*FOIG_15013*), and complemented transformant (cΔ*FOIG_15013*). (**B**) Conidial production of the tested strains. (**C**) Symptom of necrosis caused by the tested strains on the surface of tomato fruits. (**D**) Statistical analysis of the lesion size on the surface of tomato fruits. (**E**) Disease severity analyzed by percentage of amount of diseased host banana seedling with different disease grade. (**F**) Disease index. Three Δ*FOIG_15013* mutants, three complemented strains (cΔ*FOIG_15013*) and the WT strain XJZ2 were used for the pathogenicity test. A total of 30 banana seedlings were used for every tested strain. Duncan’s multiple range test was used for statistical analysis and different capital letters indicate significant difference at the level of α = 0.05. Error bars indicate S.D. (n = 3). (**G**) Hypersensitive response induced by transient expression of FOIG_15013 in *N*. *benthamiana* leaves. Leaves of *N*. *benthamiana* were infiltrated with *Agrobacterium tumefaciens* carrying pBIN::GFP-*FOIG_15013*. Photographs were taken at 2 days post-agroinfiltration. INF1 and GFP were used as positive and negative control, respectively. (**H**) Transcript levels of defense responsive genes induced by transient expression of FOIG_15013 in *N*. *benthamiana*. The constitutively expressed gene *NbEF1α* was used as internal reference [33]. Different letters indicate significant difference at the level of α = 0.01. Error bars indicate S.D. (n = 3).

In order to clarify whether *milR-87* regulates *Foc* pathogenesis through modulating *FOIG_15013* expression, we generated the Δ*FOIG_15013* mutants and corresponding complemented transformants (S6 Fig) and characterized them in growth and pathogenicity. The results showed that, the Δ*FOIG_15013* mutants grew faster and produced significantly more conidia than the WT strain XJZ2 when cultured on PDA plate. While the gene complemented transformants restored the phenotype (Fig 8A and 8B). Pathogenicity tests showed that the Δ*FOIG_15013* mutants were more virulent than the WT, while the complemented transformants exhibited a pathogenicity which is similar to that of the WT (Fig 8C–8F). These results indicate that *milR-87* may promote *Foc* pathogenicity via suppression of *FOIG_15013* expression, which is a negative regulator of *Foc* mycelial growth and virulence.

Hypersensitive response (HR), a form of programmed cell death at the site of pathogen infection, is an important symbol of plant immune activation [33]. To test the effect of FOIG_15013 on host plant immunity, we transiently expressed the *milR-87* and its target gene *FOIG_15013* in the leaves of *Nicotiana benthamiana* following the established method [18,33]. Compared to the GFP control, transient expression of FOIG_15013 caused obvious leaf yellowing in *N*. *benthamiana* at 48 hours post-agroinfiltration (hpa), while positive control of INF1 caused typical leaf necrosis at the same condition (Fig 8G). And expression of FOIG_15013 could induce significant up-regulation of some pathogenesis-related marker genes including the *PR-1*, *PAL4*, *LOX*, and Osmotin coding gene [34,35], but not induce up-regulation of HR-related gene the *HIR1* [36] and ethylene response gene the *ERF1* [37]. In contrast, co-expression of *milR-87* with FOIG_15013 significantly down-regulated these marker genes expression (Fig 8H). The results indicate the FOIG_15013 activates general defense responses in *N*. *benthamiana*, while *milR-87* suppresses these defense responses likely by silencing the expression of *FOIG_15013*.

## Discussion

Most fungal genomes encode more than one AGO proteins. The *N*. *crassa* genome has two, *M*. *oryzae* three, *C*. *parasitica* four, and members within the *F*. *oxysporum* species complex produce two to five AGO proteins [12,15,28]. Eucaryotic AGO proteins are characterized by a bilobal architecture, one contains the N-terminal and PAZ domains, and the other contains the MID and PIWI domains [38]. The *Foc* genome encodes two AGO proteins, both containing conserved PAZ and PIWI domains. Phylogenetic analysis (Fig 1B) showed the FoQDE2 proteins derived from different *formae specialis* were closely clustered in one group, suggesting that FoQDE2 proteins are well conserved and the FoQDE2 is orthologous to QDE2 proteins of *N*. *crassa*, *F*. *graminearum*, and *M*. *oryzae*.

In *F*. *oxysporum*, several genes related to conidiation were reported [29–32]. Here we selected six of them to verify the function of FoQDE2 in conidial production. qRT-PCR analysis showed that, except for *brlA* and *Htf1*, the transcription of the other four conserved genes (*abaA*, *wetA*, *FoNIIA*, and *StuA*) related to conidiation in *Fusarium* or other fungi was down-regulated in Δ*FoQDE2*. The transcript levels of only two of these genes (*FoNIIA* and *StuA*) were restored to the WT level in the complementation transformant. Previous studies have shown that a nitrite reductase coding gene, *FoNIIA*, is significantly up-regulated during conidiation compared with during vegetative growth in *F*. *oxysporum*, likely under regulation of a transcription factor Ren1 [29]. *StuA* is involved in the formation of macroconidia in *F*. *oxysporum* and is also related to spore development and pathogenicity in *F*. *graminearum* [30,39]. In our phenotypic analysis, conidiation of Δ*FoQDE2* was markedly lower than that of the WT, suggesting that *FoQDE2* could affect conidial production through down-regulating *FoNIIA* and *StuA* expression. In *Aspergillus* and other fungi, three tandem genes (*brlA*, *abaA*, and *wetA*) are considered to be central regulators of conidial production [31,32]. In this study, the transcript levels of two genes (*abaA* and *wetA*) were increased in the complementation transformant, but were not restored to the WT level, suggesting that the decreased conidiation of Δ*FoQDE2* was not directly related to this central regulatory pathway. It is unknown whether the reason for the reduced conidiation of the Δ*FoQDE2* mutant is related to the FoQDE2-dependent milRNAs.

Previous research showed that the AGO protein FgAgo1 and Dicer protein FgDicer2 function in RNAi-mediated gene silencing in *F*. *graminearum* [40]. Deletion of either of the two genes did not affect the growth and pathogenicity of *F*. *graminearum* [40]. Our results demonstrate that, compared with WT, the Δ*FoDCL1* and Δ*FoDCL2* mutants have no differences in the tested phenotypic traits, including mycelial growth and sensitivity to H_2_O_2_. The results are consistent with those reported in *F*. *graminearum* [41,42], but not the same as in *Va*. *mali* [43]. In our study, the phenotype of the Δ*FoQDE2* is completely different from that of *F*. *graminearum*. The differences in pathogenicity of *Foc* and *F*. *graminearum* may be caused by differences in infection sites. *Foc* infects the host through roots, whereas *F*. *graminearum* damages the host panicles.

Currently five types of milRNA biosynthetic pathways have been reported in fungi, four reported in *N*. *crassa* and one reported in *Ve*. *dahliae* [12,17]. Among them, *milR-1* and *milR-2* produced by the two biosynthetic pathways, respectively, are dependent on the AGO protein QDE2 in *N*. *crassa* [12]. Maturation of *milR-1* milRNAs, the most abundant milRNAs, requires the AGO protein QDE-2, Dicer, and QIP [24]. In this study, transcript level of *milR-87* was significantly lower in Δ*FoQDE2* and Δ*FoDCL2* mutant, compared with that of the WT. The result indicates *milR-87* is a FoQDE2-dependent milRNA and belongs to the *milR-1* type of milRNA.

We identified a target gene, *FOIG_15013*, of *milR-87*. *FOIG_15013* encodes a glycosyl hydrolase, shared high sequence similarity with 4-*O*-α-L-rhamnosyl-β-D-glucuronidase (FoBGlcA), a novel enzyme of *F*. *oxysproum* [44] and β-D-glucuronidase (AcGlcA79A) of *Acidobacterium capsulatum* [45]. These hydrolytic enzymes are secreted during plant-pathogen interactions and could hydrolyze D-glucuronic acid (GlcA) or 4-*O*-Me-GlcA residues at the Arabinogalactan proteins (AGPs) side-chain terminals [46,47]. It has been proposed that Arbinogalactans (AGs) are structural components of the plant cell wall and they are associated with proteins forming AGPs. Degradation products of AGPs generated by hydrolytic enzymes of pathogens could function as damage-associated molecular patterns (DAMPs) eliciting the plant defense response [48]. Therefore we speculate that product of *FOIG_15013* may secrete out and act as an avirulence effector, and likely activates the host defense responses during the infection of *Foc*. Pathogenesis-related proteins (PRs) of plant are usually activated by different biotic and abiotic stresses, including pathogen infection, to confer plant resistance [49,50]. Examples of PRs include the PR-1 and PAL4, involved in the salicylic acid (SA)-related defense pathways in *N*. *benthamiana* [50,51], the Osmotin belonging to PR-5 family and involved in the plant defense against various pathogens [52], and 9-Lipoxygenase (9-LOX) involved in activating local defense against pathogen [35,53]. In this study, heterologous transient expression of *FOIG_15013* in *N*. *benthamiana* induced significant up-regulation of these aforementioned resistant marker genes. In contrast, co-expression of *milR-87* suppressed the resistant marker genes’ expression. These results support that the *FOIG_15013* encoding glycosyl hydrolase of GH-79C family may act as an avirulence effector, thus negatively regulates *Foc* pathogencity, and the *milR-87* could suppress the expression of *FOIG_15013* to facilitate infection to the host plant. Future investigation is needed to further elucidate the hydrolase activity of FOIG_15013 and interaction between the enzyme and plant resistant marker genes expression.

Some milRNAs, found in phytopathogenic fungi *Ve*. *dahliae* and *Va*. *mali*, regulate the fungal pathogenicity by targeting their own virulent genes. For example, the *VdmilR1*, which is independent of Dicer and AGO proteins, regulates fungal virulence at the later stage of inoculation by suppressing a virulence gene (*VdHy1*) expression through increasing histone H3K9 methylation [17]. A *Vm-milR37*, exclusively expressed in the mycelium, is involved in pathogenicity by targeting a glutathione peroxidase coding gene *VmGP*, which contributes to the oxidative stress response [18]. In the study, a milRNA (*milR-87*), firstly identified in *Foc* through sRNA sequencing, contributes to the fungal virulence during the early infection stages (24–96 hpi) by targeting its own glycosyl hydrolase coding gene. So it is different from the reported milRNA found in *Ve*. *dahliae* and *Va*. *mali*.

Previous studies showed that aggressive fungal pathogens such as *Botrytis* and *Verticillium* can take up external sRNAs and double-stranded RNAs (dsRNAs) [19,54]. Applying sRNAs or dsRNAs that target *Botrytis* milRNA synthetic genes *BcDCL1* and *BcDCL2* on the surface of fruits, vegetables, and flowers significantly inhibits gray mold disease [19]. In our study, the virulence of *Foc* to banana leaves was significantly reduced by using exogenous siRNAs, a kind of dsRNAs, to suppress and interfere with the synthesis of *milR-87*, resulting in reduced disease lesion formation mimicking loss of *miR87* function. This result suggest that *milR-87* could be used as an effective anti-fungal target for developing the new and environment-friendly fungicides against banana *Fusarium* wilt.

This study provided the undisputable evidence to demonstrate the contribution of *milR-87* in promoting the virulence of *Foc* during the early infection stage. Furthermore, a novel glycosyl hydrolase acting as an avirulence effector was identified as the target of *milR-87* to activate general host defense response. And to our knowledge, it is the first report of avirulence effector targeted by fungal milRNA. The results indicate that the milRNA could silence effector coding gene expression to evade the activation of host defense response and ingeniously regulate pathogenicity of *F*. *oxysporum* f. sp. *cubense*.

## Materials and methods

### Fungal strains and culture conditions

The *F*. *oxysporum* f. sp. *cubense* tropical race 4 strain XJZ2, isolated from Guangdong Province in China [55], was used as the WT for fungal transformation, gene knockout, and milRNA overexpression experiments. All fungal strains were cultured on potato dextrose agar (PDA) for conidiation and mycelial growth. Conidiation was induced as described in our previous study [56]. After a 5-day culture on PDA plates, the colony morphology of the tested strains was recorded. Each experiment was repeated three times independently.

### Oxidative stress test

To test the sensitivity of strains to hydrogen peroxide, a conidial suspension of each strain was spotted on MM or MM supplemented with H_2_O_2_ (2 to 3 mM) and cultured at 28°C. The colony diameter was measured after 4 days’ incubation.

### Generation of deletion mutants and gene complementation transformants

Deletion mutants of three RNAi-related genes (*FoQDE2*, *FoDCL1*, and *FoDCL2*) in *Foc* were created using a conventional target gene replacement method through homologous recombination. For *FoQDE2* complementation, a 1,713-bp fragment including the promoter region and ORF of *FoQDE2* was cloned into the backbone vector pEX-Zeocin. The Δ*FoQDE2*-6 mutant was transformed with the complementation vector by PEG-mediated transformation as described previously [56]. For *FOIG_15013* gene complementation, the fragment with promoter and ORF was firstly cloned into the vector pKNT-G418 (donated by Dr. YZ Yun from Fujian Agriculture and Forestry University), then was transformed into the Δ*FOIG_15013*–19 mutant.

### Nucleic acid manipulation, small RNA detection and qRT-PCR analysis

Fungal genomic DNA was extracted according to a previously described method [57]. Deletion mutants were verified by PCR and Southern blot analysis according to a previous method [58]. Primers used in the study are shown in S1 Table. Total RNA was extracted using Trizol reagent (Invitrogen, USA) according to the manufacturer’s protocol. To detect target gene expression, qRT-PCR was carried out as described previously [57]. At least three independent experiments were performed by using three biological replicates.

For detection of small RNAs, a total of 40–60 μg total RNA was separated in a 15% urea-polyacrylamide gel, and transferred to Amersham Hybond-N^+^ membrane (GE Healthcare, USA). The probes were labelled with biotin using Biotin 3´ End DNA Labeling Kit (Thermo scientific, USA). Hybridization signals were detected by Chemiluminescent Nucleic Acid Detection Module (Thermo scientific, USA). Signal intensity was quantified using Image Lab 6.1.0 software (BIO-RAD, USA).

An All-in-One miRNA qRT-PCR Detection Kit (GeneCopoeia, Rockville, MD) was used for milRNA expression analysis [13]. Briefly, a PolyA tail was added to total RNAs using PolyA polymerase (NEB, USA). The RNA was then reverse-transcribed with an oligo-dT adaptor. MilRNA expression was detected using qRT-PCR, as described above, except that the reference gene was replaced by snRNA U4 in *Foc*. At least three biological replicates for each sample were performed.

For small RNA expression verification, reverse transcription-PCR were used in the study [42]. Briefly, cDNA was reverse-transcribed from the RNAs added with a PolyA tail with an oligo-dT adaptor. Reverse transcription-PCR was set with 20–26 cycle to avoiding nonspecific amplification.

### Small RNA library construction and sequencing

Total RNAs of the WT strain XJZ2 and *FoQDE2* deletion mutant Δ*FoQDE2* were extracted as described above. RNA integrity was assessed and quantified with a Bioanalyzer 2100 (Agilent, USA) and only qualified RNA samples (RNA integrity number, RIN: 7–10) were used for sRNA library construction. Three different samples of every strain were used for sRNA library preparation and sRNA sequencing. Small RNA libraries were prepared using NEB Next Small RNA Library Prep Set for Illumina (NEB, USA) according to the manufacturer’s protocol. Briefly, total RNAs were reverse-transcribed and indexed. Then, cDNAs were separated on a 6% polyacrylamide gel and cDNAs ranging from between 140 and 160 nt, corresponding to 20–40 nt sRNAs, were cut and recycled. The size and concentration of the sRNA sequencing libraries were assessed again using Bioanalyzer 2100. Three sRNA libraries from the WT fungus and two from the Δ*FoQDE2* mutant were qualified for sRNA sequencing. Sequencing was performed on an Illumina MiSeq platform at the University of Massachusetts, Amherst using MiSeq Reagent Kit v2 for single-end reads at a length of 50 bp.

### Small RNA data analysis and milRNA prediction

Raw sequencing data were trimmed and analyzed using CLC Genomics workbench software v10.1 (CLC bio) and only reads with a read count number larger than 10 and length between 16 and 40 nt were kept for further analysis. The *Foc* II5 (TR4 strain) genome released by the Broad Institute was used as a reference sequence. To examine the origin of the sRNA, reads mapped to the *Foc*II5 genome were collected and sequentially mapped to the rRNA, tRNA, snRNA, snoRNA, repeat, exonic, intronic, and 3′ and 5′ UTRs regions of coding genes, and intergenic regions (the region 1000 nt upstream and 1000 nt downstream of genes). To reduce false positive sRNAs induced by mRNA degradation, only reads mapped to the UTR, intron, and intergenic regions were used for sRNA length distribution and sRNA locus identification. Overlapping and adjacent reads were grouped and only sequences with a consensus length of less than 300 nt were selected and uploaded to the RNAfold web server (http://rna.tbi.univie.ac.at//cgi-bin/RNAWebSuite/RNAfold.cgi) for RNA secondary structure prediction.

To identify FoQDE2-dependent sRNAs and their loci, reads from the WT and Δ*FoQDE2* were compared to each other. Read counts were normalized by calculating their transcripts per million (TPM) value. After normalization, log2 ratios were calculated using the TPM value from two Δ*FoQDE2* mutants and three WT XJZ2 strains. Only sRNAs with log2 ratios of less than –1 or greater than 1 and a *p* value of less than 0.05 were considered as significantly expressed. The sRNAs, which were significantly down-regulated in Δ*FoQDE2* and their precursors with a typical secondary stem and loop structure were considered as FoQDE2-dependent milRNAs. For all predicted and differentially expressed milRNAs, transcript levels were verified by reverse transcription-PCR analysis.

### Generation of the milRNA deletion mutants and overexpression transformants

The milRNA deletion mutants were created by replacing the precursor (about 300 bp) of milRNA through homologous recombination as functional gene deletion we previously described [56]. The vector pSilent-1 was donated by Dr. Hitoshi Nakayashiki for milRNA overexpression. The milRNA precursor was amplified from the *Foc* genome and then cloned into the pSilent-1 vector through digestion with *Hin*dIII and *Kpn*I. The inserted fragment was verified by sequencing and the correct vector was used for transformation of the WT strain XJZ2 of *Foc*. Transformants were identified by PCR with primers given in the S1 Table.

### Transfection of siRNAs and inhibitor to suppress the production of *milR-87* in *Foc*

Protoplast preparation was performed as described previously [56]. Double-strand siRNAs (siRNA-1 and siRNA-2) and single-strand inhibitor against the precursor sequence of *milR-87*, as well as a random sRNA sequence (24 nt) serving as a negative control (NC) were designed and synthesized by General Biosystems company. According to the protocol provided by transfection reagent, a total of 100 nM siRNA oligos or inhibitor was transfected into the prepared protoplast of *Foc* using lipofectamine 2000 (Invitrogen). After two days culture, conidia suspensions of the transfected strains and positive control of the Δ*milR-87* mutant, as well as non-transfected strain of CK were inoculated on the banana leaves. After three days, the lesions on banana leaves were recorded and measured for accessing virulence of different treatments. The total RNAs of transfected strains cultured at 28°C for 7 days were extracted for milRNA detection.

### Fungal invasion assays and pathogenicity test

The ability of mycelia to penetrate cellophane and the invasive growth on tomato fruit surfaces were compared between WT and the mutants according to a previous description [56]. The pathogenicity on banana seedlings was assessed as described previously [5]. A total of 30 banana plantlets were used for each treatment. The severity of internal symptoms was recorded according to the disease grade [5], and disease indexes were calculated for disease severity assessment.

### Statistical analysis

In this study, a Student’s *t*-test was used for significance analysis of two samples. While a Duncan’s multiple range test was used to analyze the data of multiple samples at the both levels (α = 0.05 and α = 0.01). And different letters indicated significant difference.

### MilRNA target gene identification and transient expression in *Nicotiana benthamiana*

Based on the sequence of *milR-87*, its target genes in *Foc* genome were predicted using psRNATarget online software. In which the genes significantly up-regulated in the mutants Δ*milR-87* and Δ*FoQDE2* were screened by qRT-PCR and selected for target gene identification. According to the target site of the milRNA, a GFP marker gene was fused to the 3 ’terminal of the candidate target gene. And a point-mutation of the *milR-87* targeted site was introduced into the WT. The GFP fluorescence intensity representing the expression of the target gene was accessed by confocal microscopy. Furthermore, the expression of GFP and target genes was quantified by qRT-PCR.

Transient expression vector was constructed according to the method described by [33]. Briefly, precursor of the *milR-87* and *FOIG_15013* were introduced to vector pBIN:GFP. The recombinant vectors were transformed into *Agrobacterium tumefaciens* GV3101. For transient expression, transformed *A*. *tumefaciens* cultures were injected into *N*. *benthamiana* leaves [14]. After 48 h, the injected leaves were photographed and then harvested for detecting mRNA and protein levels of the FOIG_15013, as well as transcript level of resistant marker genes described previously in *N*. *benthamiana* [35,36,50–52].

## Supporting information

S1 Table Primers used in this study. (XLSX)

S2 Table Daily colony diameters of tested isolates cultured on PDA plates. (XLSX)

S1 Fig Verification of the *FoQDE2* deletion and complemented mutants by PCR and Southern blot analysis. (**A**) Schematic diagram of *FoQDE2* gene deletion and complementation. Short arrows in the figure show the primer sites in the study. (**B**) PCR identification of the *FoQDE2* deletion mutants. M, DL5000 DNA ladder purchased from TAKARA; 1–6, the different *FoQDE2* gene deletion mutants; WT, wild type strain XJZ2 of *Foc*; ddH_2_O, negative control. (**C**) Southern blot analysis with probes of the Hygromycin resistance gene fragment and the respective left border fragments of *FoQDE2*, *FoDCL1* and *FoDCL2*. WT, indicates wild type strain XJZ2 of *Foc*; 1–6, the different *FoQDE2* gene deletion mutants; 7–10, the different *FoDCL1* gene deletion mutants; 11–14, the different *FoDCL2* gene deletion mutants. Genomic DNA was digested by *Hind*III overnight, separated in a 0.8% agarose gel, blotted onto a N^+^ nylon membrane, and hybridized with the Dig-labeled HYG probe amplified with the primer pair HYG-F/HYG-R and the LB probe amplified with the primer pair P1/LB-R. (**D**) PCR identification of the *FoQDE2* complemented transformants. M, DL2000 DNA ladder purchased from TAKARA; 1–4, the different *FoQDE2* complemented transformants; 5, the WT strain; 6, the *FoQDE2* gene deletion mutant; 7, complimentary vector DNA as positive control; 8, ddH_2_O as negative control.
(TIF)

S2 Fig Gene expression in the tested strains and statistical analysis of mycelia tolerance to oxidative stress. (**A**) Relative transcript levels of *FoQDE2*, *FoDCL1*, and *FoDCL2* were examined by quantitative real-time PCR (qRT-PCR) analysis in the WT strain and their corresponding deletion mutants. (**B**) Relative transcript levels of *FoQDE2* in the WT strain, *FoQDE2* deletion mutant (Δ*FoQDE2*) and *FoQDE2* complemented transformants (cΔ*FoQDE2*-1 and cΔ*FoQDE2*-2). (**C**) Expression patterns of the conidial production-related genes in the WT strain XJZ2, the Δ*FoQDE2* mutant and complimented strain cΔ*FoQDE2*-1. (**D**) Mycelial tolerance to oxidative stress was measured by cultured the tested strains on MM with 0, 2, and 3 mM H_2_O_2_. The colony diameters of the tested strains were measured. And a Duncan’s multiple range test was used to assess significant differences. Different letters indicate the significant difference at the level of α = 0.01. Error bars indicate S. D. (n = 3).
(TIF)

S3 Fig Detection of sRNAs differentially expressed in the WT stain XJZ2 and the Δ*FoQDE2* mutant by reverse transcription-PCR. (**A**) Detection results of down-regulated sRNAs predicted in the Δ*FoQDE2* mutant compared to the WT. Three milRNAs loci showed in red were predicted to form stem-loop structure. (**B**) Detection results of up-regulated sRNAs predicted in the Δ*FoQDE2* mutant compared to the WT.
(TIF)

S4 Fig Verification of the *milR-87* deletion and overexpression mutants by PCR, qRT-PCR and Northern blot. (**A**) Schematic diagram for deletion and overexpression of milRNA (*milR-87*) in *Fusarium oxysporum* f. sp. *cubense* (*Foc*). (**B**) PCR identification of the *milR-87* deletion mutants. M1 is DL5000 DNA ladder. (**C**) PCR identification of the *milR-87* overexpression mutants. M2 is DL2000 DNA ladder. (**D**) qRT-PCR detection of m*ilR-87* in different mutants of *Foc*. (**E**) Northern blot analysis of *milR-87* in the different mutants of *Foc*.
(TIF)

S5 Fig Identification of *milR-87* target gene in *Fusarium oxysporum* f. sp. *cubense* (*Foc*). Relative transcript levels of the different target genes predicted online were examined by qRT-PCR in the WT strain XJZ2, the Δ*milR-87* mutant and the Δ*FoQDE2* mutant of *Foc*.
(TIF)

S6 Fig PCR identification of the *FOIG_15013* deletion mutants and complimented transformants of *Fusarium oxysporum* f. sp. *cubense* (*Foc*). (**A**) PCR identification of the *FOIG_15013* deletion mutants. (**B**) PCR identification of the *FOIG_15013* complimented transformants. M, DL5000 DNA ladder; XJZ2, the WT strain of *Foc*; Δ*FOIG_15013–*9/-10/-19, the different *FOIG_15013* gene deletion mutants; cΔFOIG_15013-29/-39/-51, the different *FOIG_15013* complimented transformants; ddH_2_O, negative control. (**C**) and (**D**) Relative expression of *FOIG_15013* in the tested strains and the WT strain of *Foc*. Gene expression values in different strains were normalized to that of the WT strain. Error bars indicate S.D. (n = 3). Different letters mean the significant difference at the level of α = 0.01.
(TIF)
