# Correction: FoQDE2-dependent milRNA promotes *Fusarium oxysporum f*. sp. *cubense* virulence by silencing a glycosyl hydrolase coding gene expression

**DOI:** 10.1371/journal.ppat.1011292

**Published:** 2023-03-28

**Authors:** Minhui Li, Lifei Xie, Meng Wang, Yilian Lin, Jiaqi Zhong, Yong Zhang, Jing Zeng, Guanghui Kong, Pinggen Xi, Huaping Li, Li-Jun Ma, Zide Jiang

In the Funding section, the grant number from the funder The National Science Foundation is listed incorrectly. The correct grant number is: 31871911.

## References

[1] Li M, Xie L, Wang M, Lin Y, Zhong J, Zhang Y, et al. (2022) FoQDE2-dependent milRNA promotes Fusarium oxysporum f. sp. cubense virulence by silencing a glycosyl hydrolase coding gene expression. PLoS Pathog 18(5): e1010157. 10.1371/journal.ppat.1010157 35512028 PMC9113603

