# Supplementary figures and images for: FoQDE2-dependent milRNA promotes Fusarium oxysporum f. sp. cubense virulence by silencing a glycosyl hydrolase coding gene expression
Source: PLoS Pathog. 2022 May 5;18(5):e1010157. doi: 10.1371/journal.ppat.1010157 (PMC9113603; doi:10.1371/journal.ppat.1010157)

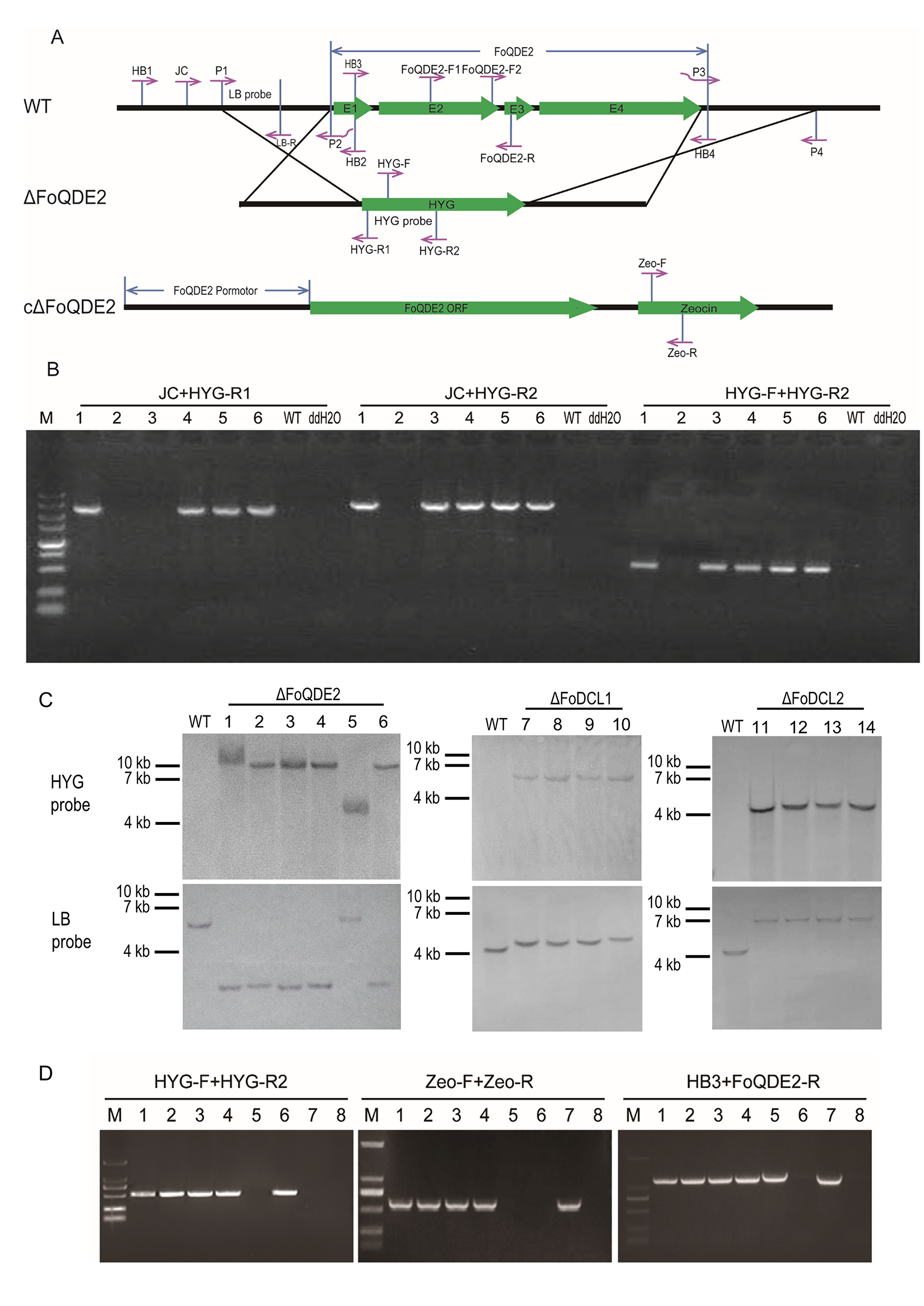

Supplement: S1 Fig — (A) Schematic diagram of FoQDE2 gene deletion and complementation. Short arrows in the figure show the primer sites in the study. (B) PCR identification of the FoQDE2 deletion mutants. M, DL5000 DNA ladder purchased from TAKARA; 1–6, the different FoQDE2 gene deletion mutants; WT, wild type strain XJZ2 of Foc; ddH2O, negative control. (C) Southern blot analysis with probes of the Hygromycin resistance gene fragment and the respective left border fragments of FoQDE2, FoDCL1 and FoDCL2. WT, indicates wild type strain XJZ2 of Foc; 1–6, the different FoQDE2 gene deletion mutants; 7–10, the different FoDCL1 gene deletion mutants; 11–14, the different FoDCL2 gene deletion mutants. Genomic DNA was digested by HindIII overnight, separated in a 0.8% agarose gel, blotted onto a N+ nylon membrane, and hybridized with the Dig-labeled HYG probe amplified with the primer pair HYG-F/HYG-R and the LB probe amplified with the primer pair P1/LB-R. (D) PCR identification of the FoQDE2 complemented transformants. M, DL2000 DNA ladder purchased from TAKARA; 1–4, the different FoQDE2 complemented transformants; 5, the WT strain; 6, the FoQDE2 gene deletion mutant; 7, complimentary vector DNA as positive control; 8, ddH2O as negative control. (TIF) [file ppat.1010157.s003.tif]

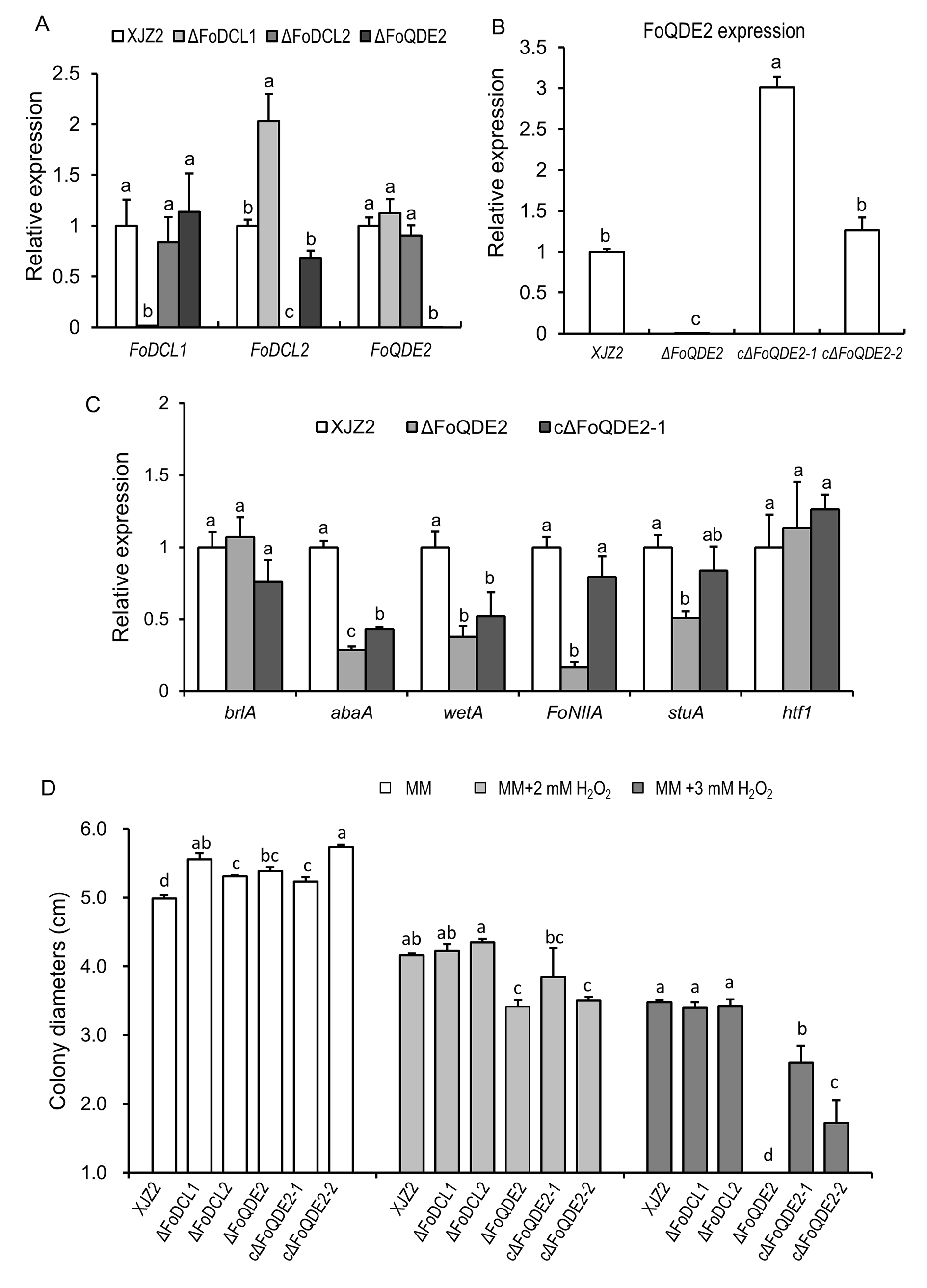

Supplement: S2 Fig — (A) Relative transcript levels of FoQDE2, FoDCL1, and FoDCL2 were examined by quantitative real-time PCR (qRT-PCR) analysis in the WT strain and their corresponding deletion mutants. (B) Relative transcript levels of FoQDE2 in the WT strain, FoQDE2 deletion mutant (ΔFoQDE2) and FoQDE2 complemented transformants (cΔFoQDE2-1 and cΔFoQDE2-2). (C) Expression patterns of the conidial production-related genes in the WT strain XJZ2, the ΔFoQDE2 mutant and complimented strain cΔFoQDE2-1. (D) Mycelial tolerance to oxidative stress was measured by cultured the tested strains on MM with 0, 2, and 3 mM H2O2. The colony diameters of the tested strains were measured. And a Duncan’s multiple range test was used to assess significant differences. Different letters indicate the significant difference at the level of α = 0.01. Error bars indicate S. D. (n = 3). (TIF) [file ppat.1010157.s004.TIF]

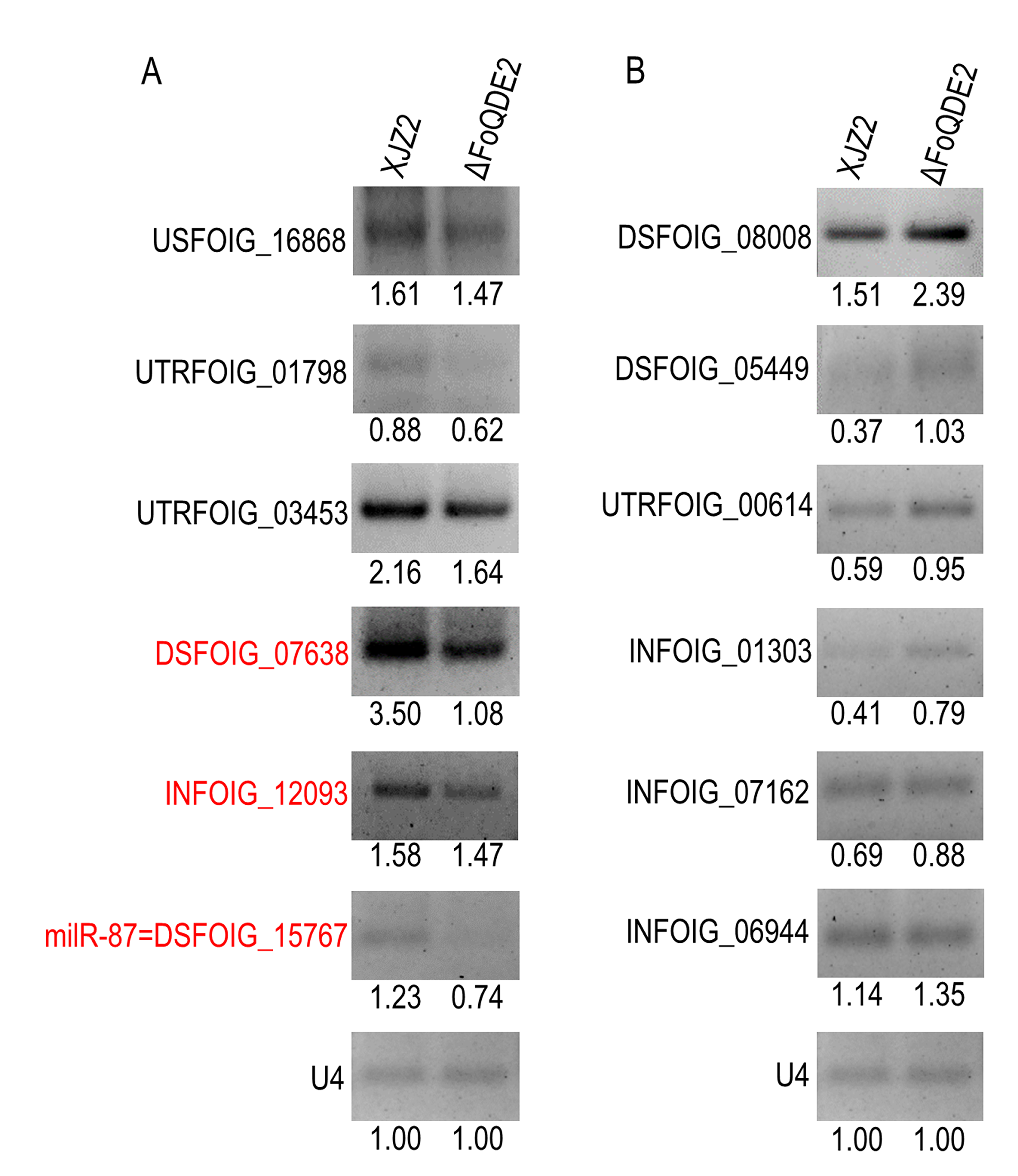

Supplement: S3 Fig — (A) Detection results of down-regulated sRNAs predicted in the ΔFoQDE2 mutant compared to the WT. Three milRNAs loci showed in red were predicted to form stem-loop structure. (B) Detection results of up-regulated sRNAs predicted in the ΔFoQDE2 mutant compared to the WT. (TIF) [file ppat.1010157.s005.tif]

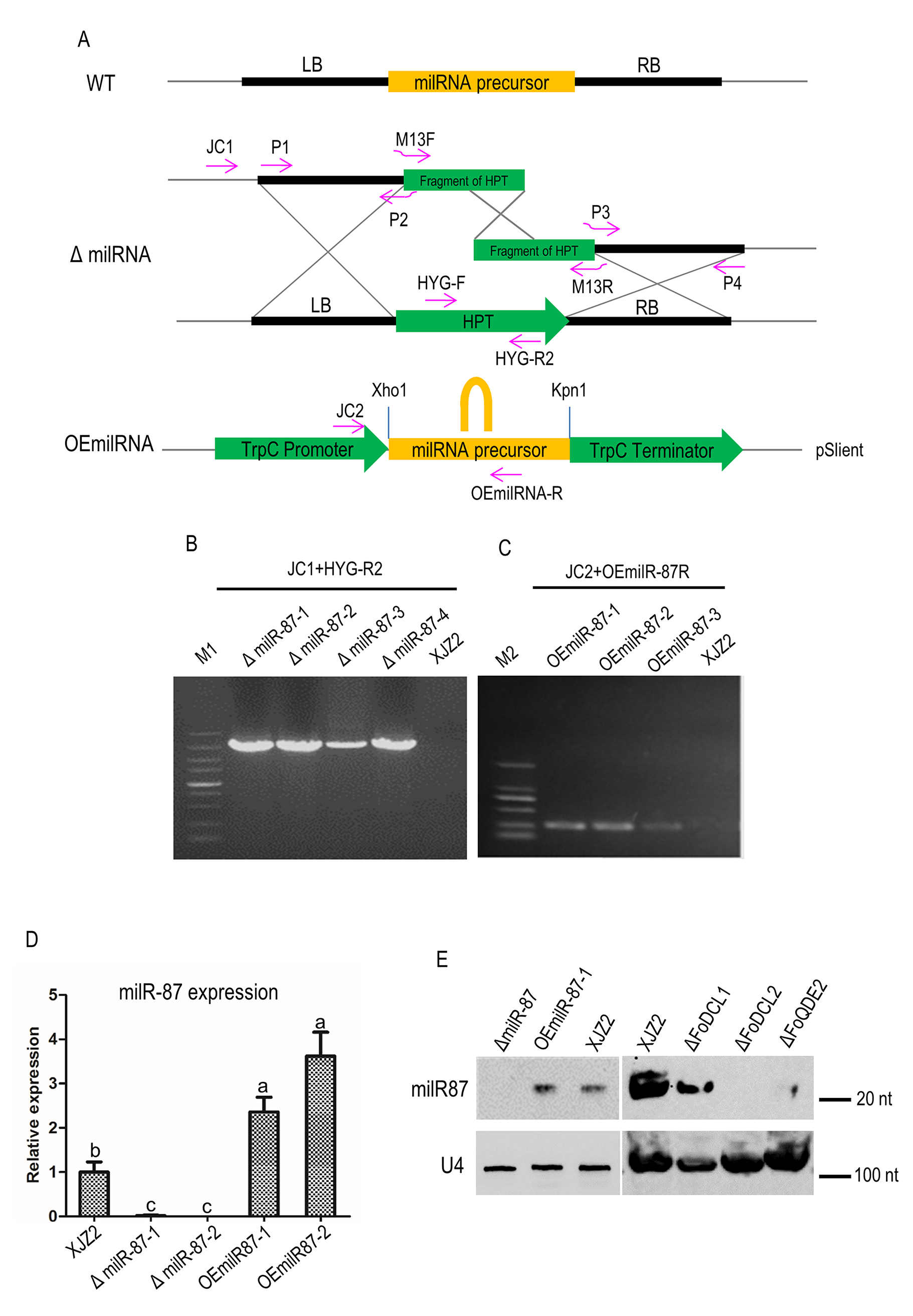

Supplement: S4 Fig — (A) Schematic diagram for deletion and overexpression of milRNA (milR-87) in Fusarium oxysporum f. sp. cubense (Foc). (B) PCR identification of the milR-87 deletion mutants. M1 is DL5000 DNA ladder. (C) PCR identification of the milR-87 overexpression mutants. M2 is DL2000 DNA ladder. (D) qRT-PCR detection of milR-87 in different mutants of Foc. (E) Northern blot analysis of milR-87 in the different mutants of Foc. (TIF) [file ppat.1010157.s006.tif]

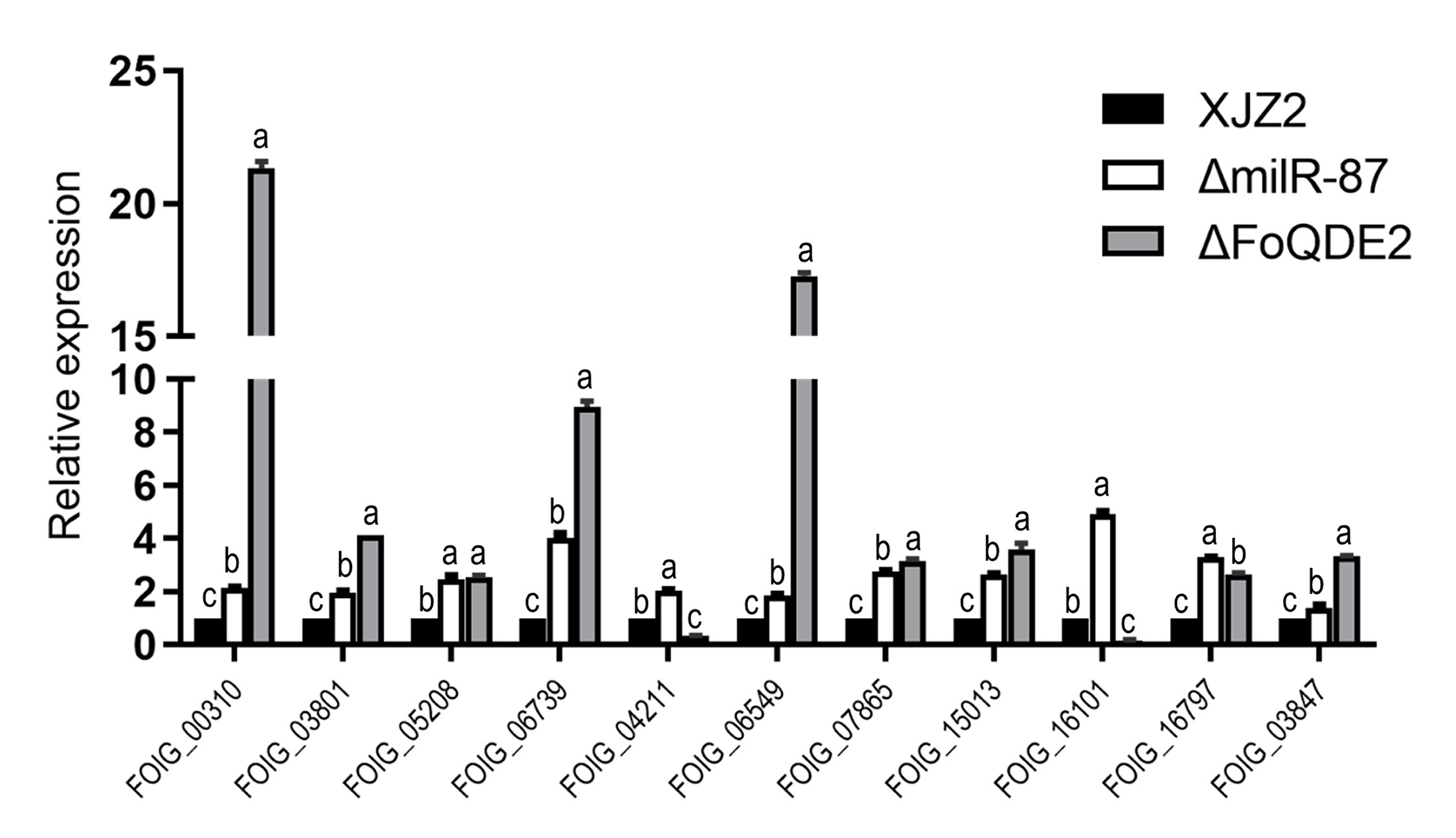

Supplement: S5 Fig — Relative transcript levels of the different target genes predicted online were examined by qRT-PCR in the WT strain XJZ2, the ΔmilR-87 mutant and the ΔFoQDE2 mutant of Foc. (TIF) [file ppat.1010157.s007.tif]

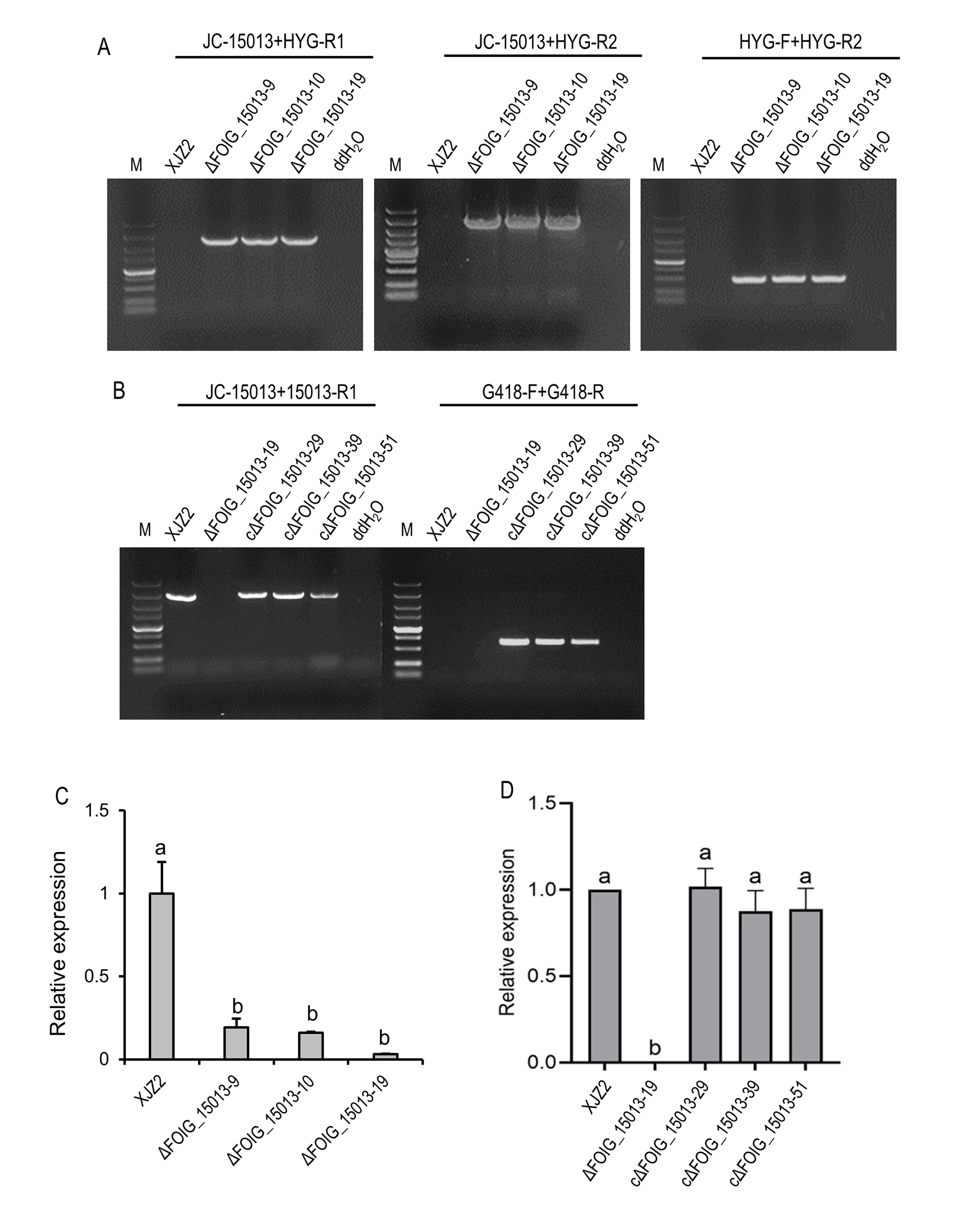

Supplement: S6 Fig — (A) PCR identification of the FOIG_15013 deletion mutants. (B) PCR identification of the FOIG_15013 complimented transformants. M, DL5000 DNA ladder; XJZ2, the WT strain of Foc; ΔFOIG_15013–9/-10/-19, the different FOIG_15013 gene deletion mutants; cΔFOIG_15013-29/-39/-51, the different FOIG_15013 complimented transformants; ddH2O, negative control. (C) and (D) Relative expression of FOIG_15013 in the tested strains and the WT strain of Foc. Gene expression values in different strains were normalized to that of the WT strain. Error bars indicate S.D. (n = 3). Different letters mean the significant difference at the level of α = 0.01. (TIF) [file ppat.1010157.s008.tif]
